# Soluble guanylate cyclase modulation in heart failure and chronic kidney disease: mechanisms, clinical evidence, and translational boundaries

**DOI:** 10.3389/fphar.2026.1954549

**Published:** 2026-10-01

**Authors:** Wenhui Yuan, Bo Zhou, Changqing Yu

**Affiliations:** Department of Cardiology, Bishan Hospital of Chongqing Medical University, Bishan Hospital of Chongqing, Chongqing, China

**Keywords:** albuminuria, cardiorenal syndrome, chronic kidney disease, evidence synthesis, heart failure, sGC activator, soluble guanylate cyclase, vericiguat

## Abstract

Heart failure (HF) and chronic kidney disease (CKD) frequently coexist and amplify one another through hemodynamic, neurohormonal, inflammatory, oxidative, and microvascular mechanisms. Impaired nitric oxide (NO)–soluble guanylate cyclase (sGC)–cyclic guanosine monophosphate (cGMP)–protein kinase G (PKG) signaling represents a biologically plausible component of this cardiorenal interaction. However, clinical translation of sGC modulation requires careful interpretation because sGC stimulators and activators preferentially engage distinct biochemical states of the enzyme, and available evidence remains heterogeneous across compounds, phenotypes, and endpoint domains. This narrative review integrates mechanistic, preclinical, and clinical evidence for clinically evaluated sGC modulators, including vericiguat, riociguat, praliciguat, runcaciguat, avenciguat, and cinaciguat, with emphasis on HF–CKD translational relevance. Vericiguat demonstrated benefit in a specific population of clinically stabilized patients with symptomatic chronic HF, LVEF below 45%, and a recent worsening-HF event. In VICTORIA, the reduction in the composite endpoint of cardiovascular death or first HF hospitalization was primarily driven by fewer HF hospitalizations, with no statistically significant reduction in cardiovascular death alone. Conversely, the VICTOR trial was neutral for its primary endpoint in stable ambulatory HFrEF, and HFpEF studies of vericiguat or praliciguat did not improve their prespecified biomarker, health-status, or exercise endpoints. In CKD populations, runcaciguat and avenciguat have shown short-term albuminuria reductions, but these surrogate signals have not established durable kidney protection, including long-term preservation of eGFR or prevention of kidney failure. To contextualize heterogeneous evidence, we propose an evidence-interpretation framework incorporating HF phenotype and clinical state, CKD phenotype, putative sGC redox biology, and endpoint domain. This framework is intended for evidence interpretation and hypothesis generation rather than patient-level treatment selection; no validated clinical assay defines tissue sGC redox state, and no prospective treatment-by-biomarker interaction has established redox-guided selection. Future studies should prioritize phenotype-enriched HF–CKD populations, contemporary background therapy, and prespecified cardiovascular and kidney outcomes to define the clinical role of sGC modulation.

## Highlights

- sGC stimulators and activators preferentially engage distinct biochemical states of sGC and should not be considered interchangeable.
- In VICTORIA, vericiguat reduced the composite of cardiovascular death or first HF hospitalization in patients with recent worsening HF and LVEF <45%, driven mainly by fewer HF hospitalizations; VICTOR was neutral for its primary endpoint in stable HFrEF.
- Runcaciguat and avenciguat have shown short-term albuminuria reductions in CKD, but these surrogate signals do not establish durable kidney protection or benefit on HF outcomes.
- The four-axis framework separates HF phenotype and clinical state, CKD phenotype defined by KDIGO CGA, putative sGC redox biology, and endpoint domain; redox-guided treatment selection remains unvalidated.

## 1 Clinical burden, therapeutic gaps, and scope of the review

HF and CKD frequently coexist, and the combination is more than the sum of two diagnoses. Across chronic and acute HF cohorts, renal impairment is common and identifies patients with higher mortality and recurrent hospitalization rates (Beldhuis et al., 2022; Lam et al., 2026; House et al., 2019; Lala et al., 2025; Rangaswami et al., 2019; Noels et al., 2025; Smith et al., 2006; Damman et al., 2014). Meta-analyses have shown that both baseline CKD and worsening renal function are associated with adverse outcomes, although the prognostic significance of a creatinine rise depends on the degree of decongestion achieved and the presence of residual congestion (Damman et al., 2014; Mullens et al., 2009; Testani et al., 2010; Metra et al., 2012).

The interaction is bidirectional. HF can reduce renal perfusion pressure, increase renal venous pressure, activate the sympathetic nervous system and the renin–angiotensin–aldosterone system, and subject the kidney to repeated episodes of congestion and decongestion. Importantly, the adverse renal effects of venous congestion may occur even when forward flow is relatively preserved (Rangaswami et al., 2019; Mullens et al., 2009; Testani et al., 2010; Metra et al., 2012). CKD, in turn, promotes sodium retention, anemia, mineral and bone disorders, uremic toxin accumulation, systemic inflammation, oxidative stress, and vascular calcification, which collectively contribute to adverse cardiac remodeling and dysfunction (Rangaswami et al., 2019; Noels et al., 2025; Kaesler et al., 2020).

Prognostic risk in CKD cannot be adequately characterized by eGFR alone. The KDIGO cause–GFR–albuminuria (CGA) framework classifies CKD by cause, GFR category (G1–G5), and albuminuria category (A1–A3) (Stevens et al., 2024). Albuminuria adds prognostic information for cardiovascular mortality, all-cause mortality, and kidney outcomes across eGFR strata (Chronic Kidney Disease Prognosis Consortium et al., 2010; Gansevoort et al., 2011). A treatment-associated change in UACR may provide an intermediate kidney signal when interpreted in context; however, a short-term reduction in UACR does not by itself establish durable kidney protection, particularly when follow-up is brief and sustained eGFR decline or kidney failure is not assessed (Coresh et al., 2019).

The contemporary therapeutic background also matters. Foundational treatment for HFrEF includes renin–angiotensin system inhibition, including angiotensin receptor–neprilysin inhibition, evidence-based beta-blockade, mineralocorticoid receptor antagonism, and sodium–glucose cotransporter 2 inhibition (Maddox et al., 2024; Heidenreich et al., 2022; McDonagh et al., 2021). These therapies should be initiated and optimized whenever feasible, while recognizing that hypotension, hyperkalemia, changes in kidney function, frailty, and polypharmacy may constrain implementation in HF–CKD (Beldhuis et al., 2022; Maddox et al., 2024; Bergen and Spaak, 2025; Pecoits‐Filho et al., 2025). Vericiguat is considered an adjunct in selected high-risk patients with HFrEF and recent worsening HF rather than a replacement for foundational therapy (Maddox et al., 2024; Heidenreich et al., 2022).

In this review, HF–CKD denotes the coexistence of chronic HF and CKD rather than a single standardized syndrome. Acute cardiorenal syndromes are discussed only when they illuminate the pharmacology, clinical development, or safety boundaries of sGC modulation. Clinical evidence is considered across HF states and phenotypes, including recently worsening HF with LVEF <45%, stable HFrEF, HFmrEF, HFpEF, and pulmonary hypertension associated with left heart disease. Throughout, evidence is distinguished among studies explicitly enrolling HF–CKD populations, HF trials with CKD subgroup analyses, CKD studies without HF outcome assessment, and preclinical combined cardiorenal models. CKD G5 without dialysis and dialysis-dependent G5D are treated as separate evidence gaps because pharmacokinetics, hemodynamic stress, competing risks, and dialyzability may differ materially (House et al., 2019; Stevens et al., 2024).

The NO–sGC–cGMP–PKG pathway is a convergent but not exclusive component of cardiorenal disease biology. The therapeutic rationale for targeting this pathway derives from its roles in vascular tone and endothelial function, myocardial stiffness and remodeling, and glomerular microcirculation, podocyte integrity, and inflammatory and fibrotic signaling (Dubin and Shah, 2016; Carlström, 2021; Carlström et al., 2024; Numata and Takimoto, 2022; Krishnan et al., 2018). Biological plausibility, however, cannot substitute for phenotype-specific clinical evidence. The central question is therefore not whether the pathway is important, but which drug, putative target state, dose, population, and endpoint are supported by direct evidence.

This review addresses three practical questions: which findings directly support current clinical use; which findings are supported only by surrogate, pharmacodynamic, or preclinical evidence; and which HF–CKD populations require dedicated studies. The resulting framework is intended to support evidence interpretation and hypothesis generation rather than patient-level treatment selection.

## 2 Literature search, study selection, and narrative evidence classification

### 2.1 Review design and purpose

This article was conducted as a structured narrative review rather than a systematic review, scoping review, or meta-analysis. Its purpose was to integrate mechanistic, preclinical, pharmacological, clinical, and regulatory evidence relevant to soluble guanylate cyclase (sGC) modulation in heart failure (HF) and chronic kidney disease (CKD), with emphasis on phenotype specificity, endpoint hierarchy, and translational boundaries. The search and selection procedures were structured to maximize transparency, reproducibility, balance, and updateability; they were not intended to establish exhaustive retrieval, support quantitative pooling, or provide a formal study-level risk-of-bias or certainty-of-evidence assessment.

### 2.2 Information sources and final search dates

PubMed/MEDLINE was searched from database inception through 29 August 2026. ClinicalTrials.gov, the U.S. Food and Drug Administration Drugs@FDA database, and the European Medicines Agency medicines database were searched through the same date. Backward citation searching was performed from pivotal randomized trials, major clinical guidelines, pharmacology reviews, and directly relevant preclinical studies; forward citation searching was used for pivotal clinical trials and major drug-development reports. The search was updated immediately before revision to identify newly indexed studies. Complete source-specific search strings, interfaces, dates, and the purpose assigned to each source are provided in Supplementary Table S1.

### 2.3 Search concepts and language restriction

The PubMed/MEDLINE strategy combined controlled vocabulary and title/abstract terms for soluble guanylate cyclase, individual sGC stimulators and activators, HF phenotypes, CKD, albuminuria, proteinuria, cardiorenal syndromes, dialysis, and NO–sGC–cGMP–PKG biology. Named agents included vericiguat, riociguat, praliciguat, runcaciguat, avenciguat, cinaciguat, BAY 1021189, BAY 63-2521, BAY 60-4552, BAY 58-2667, BAY 41-8543, BAY 60-2770, BI 685509, HEC95468, and SK-08. No publication-date or study-design filter was applied. Only English-language full-text reports that could be reliably evaluated by the review team were included; this restriction is acknowledged as a potential source of language bias.

### 2.4 Eligibility criteria

Eligible sources included: (1) randomized or non-randomized human studies evaluating an sGC stimulator or activator in HF, CKD, cardiorenal disease, albuminuric kidney disease, or pulmonary hypertension due to left-heart disease; (2) prespecified or clinically relevant secondary analyses of pivotal trials, including kidney-function, biomarker, recurrent-event, safety, background-therapy, and treatment-effect-heterogeneity analyses; (3) phase 1 and pharmacokinetic-pharmacodynamic studies that materially informed dose selection, exposure, hemodynamic effects, organ-function boundaries, or safety; (4) observational studies, meta-analyses, guidelines, consensus statements, or regulatory documents directly relevant to HF–CKD phenotyping, endpoint interpretation, approved indications, dosing, contraindications, or safety; (5) preclinical studies directly evaluating an sGC modulator in combined cardiac-renal injury, HF, CKD, albuminuria, glomerular, podocyte, microvascular, fibrotic, or redox-relevant models; and (6) mechanistic studies directly informing NO–sGC–cGMP–PKG signaling in cardiac or renal tissue.

Sources were excluded if they addressed cGMP-related biology without direct relevance to sGC or an sGC modulator; concerned pulmonary vascular indications without contributing relevant pharmacological, safety, regulatory, or left-heart-disease context; were conference abstracts, news reports, editorials, case reports, or narrative commentaries without sufficient original data; lacked a verifiable full-text publication, registry record, regulatory document, DOI, PMID, or other stable identifier; duplicated data without providing a distinct analysis or clinically relevant endpoint; were non-English reports that could not be reliably evaluated; or were outside the cardiac, renal, or cardiorenal scope of the review. Systematic reviews and meta-analyses were used to contextualize the evidence base and identify primary studies but were not substituted for pivotal primary reports when trial-specific efficacy, safety, or numerical outcomes were discussed.

### 2.5 Record management, duplicate handling, and study selection

Records were consolidated in a master reference library. Duplicates were identified using DOI, PMID, ClinicalTrials.gov registration number, exact title, and author-year-title matching and were then manually reviewed to distinguish true duplicates from companion publications, subgroup analyses, renal-function analyses, pooled analyses, or follow-up reports from the same clinical program. All potentially eligible sources and final evidence classifications were reviewed by at least two authors (WY and BZ). Disagreements were resolved by consensus, with CY adjudicating unresolved issues. Multiple reports from the same trial were retained only when they contributed distinct information, such as the primary outcome, kidney-function trajectory, recurrent events, biomarker analyses, safety, mortality, or pooled individual-participant results. The pivotal primary trial report was treated as the principal source for confirmatory efficacy conclusions.

### 2.6 ClinicalTrials.gov and unpublished or ongoing studies

ClinicalTrials.gov was searched using the generic names and development codes of the sGC modulators listed above. Registry records were classified as recruiting or not yet recruiting, active but not recruiting, completed with posted results, completed without posted results, terminated, withdrawn, or of unknown status. Registry-only and unpublished studies were used to characterize development status, planned populations, prespecified endpoints, enrollment targets, and evidence gaps; they were not treated as equivalent to peer-reviewed efficacy evidence and were not used to support definitive claims of clinical benefit. When a completed trial had posted registry results but no peer-reviewed publication, the source was explicitly identified as an unpublished registry record and assigned lower interpretive weight. When both a registry entry and a peer-reviewed publication were available, the publication was used for efficacy and safety interpretation, while the registry was used to verify study status, prespecified endpoints, and protocol-defined analyses.

### 2.7 Integration of regulatory documents and peer-reviewed evidence

Regulatory documents from Drugs@FDA, the European Medicines Agency, and current jurisdiction-specific prescribing information were used to establish approved indications, current starting and target doses, contraindications, warnings and precautions, recognized adverse reactions, use in renal or hepatic impairment, pregnancy and drug-interaction restrictions, and regulatory or development status. Regulatory documents were not used as substitutes for peer-reviewed primary trial reports when interpreting efficacy. Where trial publications, registry records, and regulatory documents addressed different questions, they were integrated according to their evidentiary roles: the peer-reviewed trial report informed efficacy and safety estimates, the registry clarified protocol-defined endpoints and development status, and regulatory labeling defined currently approved use and dosing.

### 2.8 Data extraction and narrative evidence classification

For clinical studies, the review extracted study design and phase, population and sample size, HF phenotype and clinical state, CKD phenotype and kidney-function range, intervention and comparator, treatment duration, background therapy, primary and secondary endpoints, follow-up, efficacy findings, safety findings, regulatory status, and principal inferential limitations. For preclinical studies, extracted domains included species, disease model, sex, agent, dose, duration, cardiac findings, kidney findings, blood-pressure context, comparator design, and translational limitations.

Evidence was classified by domain rather than assigned a formal numerical score. Randomized clinical-outcome trials in directly relevant populations received the greatest weight for efficacy. Prespecified secondary analyses and pooled analyses refined interpretation but did not supersede the primary trial result. Phase 1 and phase 2 biomarker, hemodynamic, pharmacokinetic, and surrogate-endpoint studies were interpreted as developmental or mechanistic evidence. Observational and *post hoc* analyses were considered hypothesis-generating. Preclinical studies were used to establish biological plausibility, target engagement, and model-specific pharmacology but were not treated as direct evidence of clinical benefit. Clinical events, kidney clinical outcomes, surrogate endpoints, hemodynamics, biomarkers, pharmacodynamic findings, histology, and molecular outcomes were kept analytically distinct.

### 2.9 Narrative-review status and interpretive boundaries

The use of reproducible searches, explicit eligibility criteria, duplicate management, multi-author review, and a predefined narrative evidence hierarchy was intended to reduce selective emphasis and improve transparency. These procedures do not convert the article into a systematic or scoping review. No protocol was prospectively registered, no formal study-level risk-of-bias instrument or certainty-of-evidence framework was applied, and no quantitative meta-analysis was performed. The article should therefore be interpreted as a structured, clinically oriented narrative synthesis that aims to provide a current and balanced overview while preserving explicit distinctions among mechanistic, preclinical, surrogate, clinical-outcome, and regulatory evidence.

## 3 Cardiorenal pathobiology of NO–sGC–cGMP–PKG signaling

### 3.1 Hemodynamic and systemic cardiorenal crosstalk

Reduced cardiac output can compromise renal perfusion, but venous congestion is also a major determinant of renal dysfunction in decompensated HF (Rangaswami et al., 2019; Mullens et al., 2009). Elevated central venous pressure can be transmitted to the renal veins, increase renal interstitial pressure, and reduce sodium excretion and GFR (Mullens et al., 2009). Accordingly, worsening renal function during treatment should not be interpreted in isolation: its prognostic significance may differ according to the degree of decongestion achieved and whether congestion persists (Testani et al., 2010; Metra et al., 2012).

CKD adds nonhemodynamic stressors, including anemia and iron abnormalities, disordered mineral metabolism, uremic toxins, chronic inflammation, oxidative stress, and endothelial dysfunction (Rangaswami et al., 2019; Kaesler et al., 2020; Wang et al., 2025). Sodium and water retention can further increase volume load and thereby amplify congestion (Noels et al., 2025). Together, these processes define a multifactorial cardiorenal milieu rather than a single hemodynamic syndrome (Rangaswami et al., 2019; Noels et al., 2025).

### 3.2 Reduced NO bioavailability, eNOS uncoupling, and oxidative stress

Endothelial NOS-derived NO contributes to vascular homeostasis and activates sGC to generate cGMP (Carlström et al., 2024; Förstermann et al., 2012). In the kidney, constitutive NO signaling modulates renal vascular resistance, medullary perfusion, tubular sodium transport, and pressure natriuresis (Carlström, 2021; Mount and Power, 2006).

In cardiovascular and kidney disease, NO bioactivity can be reduced by impaired NOS-dependent production, endogenous NOS inhibition, and accelerated scavenging by reactive oxygen species (Carlström, 2021; Carlström et al., 2024). Asymmetric dimethylarginine (ADMA) competitively inhibits NOS and accumulates in advanced CKD and kidney failure, although DDAH-mediated degradation is quantitatively more important than renal excretion in regulating ADMA concentrations (Zoccali, 2006). BH_4_ deficiency or oxidation can uncouple eNOS, shifting electron transfer toward superoxide rather than NO generation (Förstermann and Münzel, 2006; Schulz et al., 2008). Reactive oxygen species derived from NADPH oxidases, mitochondria, and xanthine oxidase can amplify this process, while superoxide reacts rapidly with NO to form peroxynitrite and further reduces NO bioactivity (Schulz et al., 2008). Taken together, reduced NO bioactivity in cardiorenal disease reflects several interacting defects rather than a uniform state of simple “NO deficiency.”

### 3.3 sGC architecture and the redox continuum

sGC is a heterodimeric NO receptor, and the β1 H-NOX domain contains a ferrous (Fe^2+^) heme that is required for NO sensing (Derbyshire and Marletta, 2012; Liu et al., 2021). NO binding induces allosteric conformational changes that activate the catalytic domain and increase cGMP synthesis (Liu et al., 2021).

Oxidation of the heme from Fe^2+^ to ferric Fe^3+^ reduces NO responsiveness, and ferric heme can dissociate to generate heme-free apo-sGC with low activity and poor responsiveness to NO (Derbyshire and Marletta, 2012; Liu et al., 2021). These redox states provide the biochemical basis for the distinction between sGC stimulators and activators, which is considered pharmacologically in Section 4 (Liu et al., 2021; Stasch et al., 2011; Evgenov et al., 2006).

The biochemical redox distinction is not a validated clinical classifier. No validated clinical assay currently quantifies tissue sGC redox state, and no prospective treatment-by-biomarker interaction has established a redox threshold for choosing an activator over a stimulator. Because cGMP signaling is spatially compartmentalized, systemic oxidative-stress markers and circulating cGMP cannot be assumed to identify the relevant intracellular sGC redox or signaling state in cardiac or renal target tissue (Cuello and Nikolaev, 2020; Stehle et al., 2022). Accordingly, oxidative stress, advanced CKD, or proteinuria should not currently be interpreted as defining an “activator-responsive” phenotype.

### 3.4 cGMP compartmentalization and phosphodiesterase control

In cardiomyocytes, NO–sGC- and natriuretic-peptide–particulate-guanylyl-cyclase-derived cGMP signals are organized within functionally distinct intracellular microdomains (Cuello and Nikolaev, 2020; Takimoto, 2012). Experimental cardiomyocyte studies further indicate that PDE5A preferentially regulates NO-derived cGMP, whereas PDE9A preferentially regulates natriuretic-peptide-derived cGMP (Lee et al., 2015). Thus, the source and subcellular location of cGMP may influence downstream signaling, but compartmentalization alone does not predict clinical response (Cuello and Nikolaev, 2020; Lee et al., 2015).

### 3.5 Cardiac consequences: titin, calcium, hypertrophy, and fibrosis

Experiments in human donor-heart myofibers and myofibrils showed that cGMP-activated PKG phosphorylates titin and, after experimental dephosphorylation, reduces passive stiffness by approximately 10%–20% (Krüger et al., 2009). Compared with aortic stenosis and HFrEF, HFpEF myocardial biopsies showed lower PKG activity, which was associated with lower myocardial cGMP, higher myocardial nitrotyrosine, and greater cardiomyocyte passive stiffness; the elevated stiffness was reduced by *in vitro* PKG administration (van Heerebeek et al., 2012). Antihypertrophic signaling was less consistent across experimental models: PKG I activation suppressed α1-adrenergic-stimulated calcineurin–NFAT signaling, BNP induction, and cell enlargement in neonatal rat ventricular cardiomyocytes (Fiedler et al., 2002), whereas hypertrophy induced by chronic isoproterenol or transverse aortic constriction was not greater in mice lacking cGKI in cardiomyocytes than in controls (Lukowski et al., 2010).

The HFpEF paradigm proposed by Paulus and Tschöpe links comorbidity-driven systemic inflammation to coronary microvascular endothelial inflammation and oxidative stress, reduced NO–cGMP–PKG signaling, cardiomyocyte stiffness and hypertrophy, and interstitial fibrosis (Paulus and Tschöpe, 2013). HFpEF is nevertheless phenotypically heterogeneous: in a prospective cohort of 397 patients, phenomapping identified three groups with distinct clinical characteristics, cardiac structure and function, invasive hemodynamics, and outcomes (Shah et al., 2015). These findings support mechanistic plausibility but do not establish cGMP–PKG deficiency as a uniform cause of HFpEF, nor do they show that heterogeneity explains the neutral HFpEF trials of sGC stimulation. Phenotype-based enrichment remains a hypothesis for prospective testing.

### 3.6 Kidney microcirculation, podocytes, and tubulointerstitial fibrosis

Within the kidney, sGC is present in vascular and glomerular compartments; rat studies localize it to glomerular arterioles, mesangial cells, descending vasa recta, and interstitial fibroblasts (Thei et al., 2001). In murine preparations, sGC activators increase glomerular cGMP, dilate afferent and efferent arterioles, and improve renal blood flow under experimentally reduced NO signaling or ODQ exposure (Stehle et al., 2022; Wennysia et al., 2021). The implications of these acute vascular effects for filtration and long-term human kidney function remain uncertain.

NO and riociguat also increase cGMP and reduce TRPC6-mediated injury in cultured human podocytes (‘T Hart et al., 2021). In rat mesangial cells, cGMP–PKG signaling suppresses high-glucose-induced thrombospondin-1-dependent TGF-β activation and matrix expression, while increased PKG activity or sGC stimulation attenuates fibrosis in unilateral ureteral obstruction models (Wang et al., 2003; Wang et al., 2002; Cui et al., 2014; Schinner et al., 2017).

These mechanisms provide a plausible basis for changes in albuminuria, but not proof of durable renoprotection. Albuminuria change is associated with subsequent kidney-failure risk and has been evaluated as a surrogate endpoint (Coresh et al., 2019). Short-term drug-induced UACR reductions therefore require confirmation by sustained preservation of kidney function or clinical kidney outcomes.

### 3.7 Mechanistic synthesis and translational boundary

The NO–sGC–cGMP–PKG pathway plausibly connects vascular dysfunction with myocardial stiffness and remodeling and with renal microvascular and glomerular injury. Yet the supporting evidence is uneven across tissues, experimental models, and disease phenotypes, and demonstration of pathway engagement does not itself establish clinical benefit. This biology provides a rationale for evaluating sGC stimulators and activators separately, whereas efficacy and safety must be judged from agent-specific evidence in the populations and endpoints studied.

## 4 Pharmacological distinction between sGC stimulators and activators

### 4.1 sGC stimulators

sGC stimulators preferentially act on reduced, heme-containing, NO-sensitive sGC, enhancing the response to endogenous NO while also directly stimulating the enzyme when NO availability is low (Stasch et al., 2011; Evgenov et al., 2006; Follmann et al., 2013; Tawa et al., 2025). Riociguat established this pharmacological class in pulmonary vascular disease, whereas vericiguat was developed for chronic HF through optimization of metabolic stability and clearance to provide a longer duration of action compatible with once-daily oral dosing, rather than the three-times-daily regimen required for riociguat (Follmann et al., 2017).

Praliciguat entered a broader clinical-development program that included cardiometabolic disease, HFpEF, and diabetic kidney disease (Udelson et al., 2020; Hanrahan et al., 2021; Hanrahan et al., 2020). In participants with type 2 diabetes and hypertension, it was rapidly absorbed (median Tmax, 1–3 h), with a large apparent volume of distribution and an effective half-life of 39.7 h, a pharmacokinetic profile consistent with extensive tissue dispersal and once-daily dosing (Hanrahan et al., 2020).

### 4.2 sGC activators

sGC activators bind oxidized or heme-free sGC and increase cGMP independently of NO. Cinaciguat, runcaciguat, and avenciguat are representative agents, while SK-08 has recently entered early clinical evaluation as an oral activator. The biochemical distinction from sGC stimulators is well established, but its use for clinical treatment selection remains unvalidated, as discussed in Section 3.3 (Liu et al., 2021; Stasch et al., 2011; Evgenov et al., 2006; Follmann et al., 2013; Tawa et al., 2025; Stasch et al., 2015; Bénardeau et al., 2021; Reinhart et al., 2023; Gansevoort et al., 2025; Heerspink et al., 2024; Liu et al., 2026).

Cinaciguat was developed for intravenous use in acute decompensated HF. Runcaciguat and avenciguat are oral activators developed primarily for CKD, whereas human experience with SK-08 is limited to a single-ascending-dose phase 1 study in 16 healthy participants and does not establish efficacy in CKD (Gansevoort et al., 2025; Heerspink et al., 2024; Liu et al., 2026; Erdmann et al., 2013; Gheorghiade et al., 2012; Lapp et al., 2009).

### 4.3 Exposure, hemodynamics, and class boundaries

The clinical effect of an sGC modulator depends on more than the nominal drug class. Oral bioavailability, tissue distribution, peak-to-trough exposure, dosing frequency and titration, food effect, concomitant vasodilators, blood-pressure reserve, anemia, kidney function, and volume status can all influence efficacy and tolerability (Follmann et al., 2017; Hanrahan et al., 2020; Gansevoort et al., 2025; Heerspink et al., 2024; Liu et al., 2026; Erdmann et al., 2013; Gheorghiade et al., 2012; Lapp et al., 2009; Armstrong et al., 2020a). These factors also shape the therapeutic window: strong pathway engagement or hemodynamic activity may become clinically limiting when accompanied by excessive blood-pressure reduction, as illustrated by the cinaciguat program (Erdmann et al., 2013; Gheorghiade et al., 2012; Lapp et al., 2009).

Route of administration and development setting further limit cross-agent extrapolation. An agent developed for acute intravenous unloading cannot be assumed to predict chronic oral therapy, just as an albuminuria-focused CKD program cannot establish HF outcome efficacy. Pharmacological class therefore provides a mechanistic starting point, but clinical interpretation remains specific to the individual compound, its exposure profile, the population studied, and the endpoint assessed.

## 5 Preclinical evidence and translational limitations

### 5.1 Hypertension, pressure load, and cardiorenal injury

Dahl salt-sensitive rats exposed to a high-salt diet develop hypertension, cardiac hypertrophy, inflammation, proteinuria, and renal fibrosis. Praliciguat attenuated inflammatory and fibrotic signals and reduced end-organ injury, with renal histological injury, inflammation, and fibrosis attenuated even at 1 mg/kg/day, a dose with little effect on blood pressure, whereas higher doses produced clearer antihypertensive effects (Shea et al., 2020). HEC95468, another stimulator, reduced cardiac and renal injury and improved survival in a related model (Wu et al., 2025), while prolonged cinaciguat exposure improved structural and survival measures in salt-sensitive disease while significantly blunting the salt-induced rise in blood pressure (Hoffmann et al., 2015).

Taken together, these studies support pharmacological activity of sGC modulation in a combined cardiac-renal injury phenotype, but they do not establish uniformly blood-pressure-independent organ protection. None of these studies used a formal blood-pressure-matched antihypertensive control. The low-dose praliciguat findings nevertheless argue against blood-pressure lowering as the sole explanation for the renal effects in that experiment, whereas hemodynamic contributions remain difficult to separate for HEC95468 and cinaciguat. Rodent salt-sensitive disease also lacks the age, sex distribution, multimorbidity, contemporary multidrug therapy, recurrent episodes of decompensation, and competing risks of human HF–CKD.

### 5.2 Volume overload and venous congestion

In hypertensive Ren-2 transgenic rats with an aortocaval fistula, the sGC stimulator BAY 41-8543 increased urinary cGMP and improved survival. In the 210-day survival protocol, survival at day 60 of therapy was 50% in BAY 41-8543-treated rats (n = 26) versus 8% in untreated ACF TGR (n = 24), although the survival advantage attenuated after day 140. At the selected dose, plasma exposure was confirmed, while the blood-pressure reduction was early and transient, with no difference from untreated ACF TGR by day 14. The model combines pre-existing hypertension and endogenous RAAS activation with surgically induced high-output volume-overload HF and therefore does not reproduce heterogeneous chronic human HF. Moreover, cardiac function was not comprehensively characterized during the long-term survival protocol. Thus, the survival signal supports biological plausibility rather than direct inference of clinical benefit in HFrEF or HFpEF (Gawrys et al., 2023).

### 5.3 Diabetic, metabolic, and proteinuric CKD

Runcaciguat reduced proteinuria and kidney-injury markers across hypertensive, diabetic, and metabolic CKD models, with structural improvements in selected models; some effects occurred at doses with little or no sustained blood-pressure lowering (Bénardeau et al., 2021). In obese ZSF1 rats, it attenuated decline in kidney function (Kraehling et al., 2023). Single-cell analysis identified differential treatment responses, particularly in proximal tubular and stromal compartments, but activator-stimulator dose-response matching was incomplete (Balzer et al., 2023).

Avenciguat reduced proteinuria and glomerulosclerosis in ZSF1 rats on enalapril and tubulointerstitial fibrosis in obstructive nephropathy (Reinhart et al., 2023; Sharma et al., 2025). In uninephrectomized diabetic db/db mice, combination with empagliflozin further reduced albuminuria and selected renal injury/fibrotic markers (Sharma et al., 2025). Earlier ZSF1 activator studies also showed antiproteinuric and structural effects at doses without significant MAP reduction (Boustany-Kari et al., 2016).

In ZSF1 rats, praliciguat attenuated proteinuria without lowering MAP during monotherapy and reduced the expression of selected renal genes related to inflammation, fibrosis, oxidative stress, and kidney injury (Liu et al., 2020).

### 5.4 Advanced CKD and redox stress

In 5/6 nephrectomy models, sGC activation improved selected renal structural and functional measures, although proteinuria was not consistently reduced. In 5/6 nephrectomized rats on a high-salt diet, BAY 41-8543 and BAY 60-2770 lowered blood pressure to a similar extent; neither improved proteinuria or renal functional parameters, whereas the activator produced greater reductions in interstitial fibrosis and glomerulosclerosis than the stimulator. In an earlier 5/6 nephrectomy study, BAY 58-2667 improved creatinine clearance and attenuated renal structural injury but markedly lowered blood pressure and did not reduce proteinuria. These findings are consistent with, but do not validate, a redox-dependent treatment hypothesis because tissue sGC redox state was not directly established and may differ across renal compartments. These models cannot define a human eGFR threshold, predict dialysis pharmacology, or establish advanced CKD as an activator-responsive phenotype (Chen et al., 2024; Kalk et al., 2006).

### 5.5 Translational limitations

Preclinical models capture selected components of cardiorenal injury but not the heterogeneity or treatment context of human HF–CKD. Few reproduce contemporary multidrug therapy, recurrent decompensation, or dialysis. Formal BP-matched antihypertensive controls were not used in the principal studies summarized here; doses with little sustained BP effect or active comparators provide only suggestive evidence for non-hemodynamic effects. Sex-specific responses, tissue target engagement, pharmacokinetic–pharmacodynamic (PK–PD) relationships, and BP mediation remain incompletely resolved. Histology, molecular signatures, urinary biomarkers, and short-term kidney-function measures inform mechanism and dose selection but do not establish clinical cardiorenal benefit (Stasch et al., 2015; Sandner and Stasch, 2017; Zhang et al., 2026). Table 1 summarizes these limitations.

**TABLE 1:** Preclinical evidence for sGC modulation in cardiorenal disease models and major translational limitations.

Pathological module	Model/species	Agent and class	Regimen/duration	Main cardiac findings	Main kidney findings	BP/safety context	Major translational limitation
Pressure-load cardiorenal injury	Male Dahl salt-sensitive rats; high-salt diet	Praliciguat (stimulator)	1, 3, or 10 mg/kg/day; 6 weeks (Shea et al., 2020)	Attenuated cardiac hypertrophy; high-dose improvement in cardiac function and stress markers	Lower albuminuria; attenuated glomerular injury, inflammation, and fibrosis	1 mg/kg/day had little BP effect; higher doses lowered BP	No formal BP-matched control; single salt-sensitive model without contemporary multidrug background
Pressure-load cardiorenal injury	Dahl salt-sensitive rats; high-salt diet	HEC95468 (stimulator)	3 or 10 mg/kg/day; 13 weeks (Wu et al., 2025)	Reduced cardiac hypertrophy, stress markers, and fibrosis; survival improved	Lower UPCR, urinary KIM-1, serum creatinine, glomerulosclerosis, tubular injury, and interstitial fibrosis	Marked dose-dependent BP lowering; treatment groups approached control BP	Organ effects are strongly confounded by BP lowering; single-model study with no human outcome inference
Pressure-load cardiorenal injury	Male Dahl salt-sensitive rats; high-salt diet	Cinaciguat (activator)	1000 ppm in feed; 21 weeks (Hoffmann et al., 2015)	Improved survival; reduced left-ventricular profibrotic/proinflammatory markers and selected cardiac histopathology	Lower proteinuria, higher creatinine clearance, and reduced renal inflammatory/fibrotic markers	Substantially attenuated the high-salt-induced BP rise	Hemodynamic mediation remains difficult to separate from organ protection
Volume overload	Male hypertensive Ren-2 rats with aortocaval fistula	BAY 41-8543 (stimulator)	3 mg/kg/day; 1-week pharmacodynamic, 2-week BP, and 210-day survival protocols (Gawrys et al., 2023)	Improved survival; 60-day survival 50% vs. 8% untreated, with attenuation after day 140	Increased urinary cGMP; long-term renal histology limited by survivor depletion	Early transient BP reduction; no difference vs. untreated by day 14	High-output surgical model; no comprehensive cardiac-function assessment; limited clinical HF extrapolation
Hypertensive/diabetic/metabolic CKD	Multiple hypertensive, diabetic, and metabolic CKD rat models, including obese ZSF1	Runcaciguat (activator)	Model-specific oral regimens; 2–42 weeks (Bénardeau et al., 2021; Kraehling et al., 2023)	Survival and heart-injury markers improved in renin-transgenic rats; cardiac endpoints secondary overall	Reduced proteinuria and kidney-injury measures; structural or kidney-function benefits in selected models	Several effects occurred with no/modest BP lowering; low/mid ZSF1 doses lacked sustained BP reduction	No formal BP-matched control; outcomes remain predominantly surrogate, functional, or histological rather than clinical cardiorenal outcomes
Diabetic kidney disease	Obese ZSF1 rats; single-cell analysis	Runcaciguat (activator) vs. BAY-747 (stimulator)	3 mg/kg twice daily vs. 3 mg/kg once daily; 12 weeks (Balzer et al., 2023)	No direct cardiac outcome inference	Cell-type-specific responses, particularly in proximal tubular and stromal compartments; activator greater on selected readouts	Selected doses had no/minimal BP effects	Activator–stimulator dose-response matching was incomplete; direct vs. indirect renal-cell effects remain unresolved
Renal injury/fibrosis	Obese ZSF1 rats on enalapril; unilateral ureteral obstruction rats	Avenciguat (activator)	1–30 mg/kg/day for 10 weeks (ZSF1); 7 days (obstruction model) (Reinhart et al., 2023)	No direct HF-outcome inference	Reduced proteinuria and glomerulosclerosis on enalapril; reduced tubulointerstitial fibrosis in obstruction model	MAP was modestly reduced at higher ZSF1 doses vs. enalapril	Model-specific surrogate/structural endpoints; no direct HF or kidney-outcome inference
Diabetic CKD combination	Uninephrectomized db/db mice	Avenciguat + empagliflozin	6 weeks (Sharma et al., 2025)	No human HF inference	Combination further reduced albuminuria and selected renal injury/fibrotic markers relative to one or both monotherapies, depending on endpoint	Glycemic effects may contribute; BP was monitored without reported BP-related adverse effects	Short preclinical combination study; incremental clinical renal benefit requires human add-on/factorial evaluation
Diabetic nephropathy	Male obese ZSF1 rats	BI 703704 (activator)	0.3–10 mg/kg/day; 15 weeks (Boustany-Kari et al., 2016)	Cardiac endpoints not primary	Dose-dependent reductions in proteinuria and glomerulosclerosis; tubulointerstitial injury markers also improved	0.3–3 mg/kg did not significantly lower MAP; enalapril was an active comparator	No formal BP-matched design; surrogate/structural endpoints and metabolic effects limit mechanistic attribution
Diabetic nephropathy	Male obese ZSF1 rats	Praliciguat (stimulator)	1–10 mg/kg/day ± enalapril 3 mg/kg/day; 11 weeks (Liu et al., 2020)	Cardiac endpoints not primary	Attenuated proteinuria at 10 mg/kg/day; lower renal inflammatory, fibrotic, oxidative-stress, and injury-related gene expression	Praliciguat monotherapy did not affect hemodynamics; enalapril lowered BP but did not attenuate proteinuria	Suggestive, not definitive, evidence for non-hemodynamic renal effects; molecular and proteinuria endpoints do not establish clinical kidney benefit
Advanced CKD/oxidative stress	5/6 nephrectomy + high-salt rats	BAY 41-8543 (stimulator) vs. BAY 60-2770 (activator)	11 weeks (Chen et al., 2024)	Not designed for HF outcomes	Neither improved proteinuria or renal functional parameters; activator reduced interstitial fibrosis and glomerulosclerosis more than stimulator	Comparable BP lowering at week 11 (tail-cuff)	Non-clinical tool compounds; tissue sGC redox state was not directly established, so the study does not validate an activator-responsive CKD phenotype
Advanced CKD	5/6 nephrectomy rats	BAY 58-2667 (activator)	3,000 ppm in feed (∼50 mg/day); 18 weeks (Kalk et al., 2006)	Reduced left-ventricular hypertrophy and selected cardiac structural measures	Improved creatinine clearance, glomerulosclerosis, and renal fibrosis; no antiproteinuric effect	Marked BP reduction; authors considered hemodynamic mediation likely	No BP-matched control; the study could not establish BP-independent effects; older model without contemporary CKD therapy

Abbreviations: BP, blood pressure; CKD, chronic kidney disease; cGMP, cyclic guanosine monophosphate; HF, heart failure; HF–CKD, heart failure–chronic kidney disease; KIM-1, kidney injury molecule-1; MAP, mean arterial pressure; NO, nitric oxide; ppm, parts per million; sGC, soluble guanylate cyclase; UPCR, urinary protein-to-creatinine ratio.

## 6 Clinical evidence

The clinical evidence summarized below derives from distinct settings, including HF trials with CKD represented or analyzed secondarily, CKD trials without HF outcome assessment, and studies of related HF phenotypes; these evidence contexts should not be interpreted as equivalent direct evidence for HF–CKD.

### 6.1 Early HFrEF development and SOCRATES-REDUCED

SOCRATES-REDUCED was a phase 2 dose-finding trial that randomized 456 clinically stabilized patients with worsening chronic HF and LVEF below 45% to placebo or four vericiguat dose regimens for 12 weeks. The primary pooled comparison of the 2.5-, 5-, and 10-mg groups versus placebo did not significantly reduce NT-proBNP (ratio of geometric means 0.885, 90% CI 0.73–1.08; P = 0.15). A prespecified secondary analysis suggested a dose-response relationship, but because the primary analysis was neutral, this signal remained exploratory and informed subsequent phase 3 development rather than establishing clinical-outcome efficacy (Gheorghiade et al., 2015).

### 6.2 Recently worsening symptomatic HF: VICTORIA

VICTORIA was a phase 3, randomized, double-blind, placebo-controlled trial of 5,050 patients with symptomatic chronic HF, LVEF below 45%, and a recent worsening event defined by HF hospitalization within 6 months or outpatient intravenous diuretic treatment within 3 months (Armstrong et al., 2020a; Armstrong et al., 2018). Approximately 60% of participants were receiving triple therapy and about 15% an ARNI at baseline (Armstrong et al., 2020a; Pieske et al., 2019). SGLT2 inhibitor use was uncommon, so the trial could not establish the incremental effect of vericiguat when added to SGLT2 inhibitor therapy (Armstrong et al., 2020a).

Over a median follow-up of 10.8 months, the primary composite of cardiovascular death or first HF hospitalization occurred in 35.5% of the vericiguat group and 38.5% of the placebo group (HR 0.90, 95% CI 0.82–0.98; P = 0.02). HF hospitalization occurred in 27.4% versus 29.6% (HR 0.90, 95% CI 0.81–1.00), whereas cardiovascular death occurred in 16.4% versus 17.5% (HR 0.93, 95% CI 0.81–1.06). Thus, the positive primary result was driven predominantly by fewer HF hospitalizations rather than a statistically significant reduction in cardiovascular mortality (Armstrong et al., 2020a).

Secondary analyses showed that event risk was highest in patients randomized closest to the index HF hospitalization, without significant heterogeneity in treatment effect across index-event timing subgroups (Lam et al., 2021a). Higher baseline NT-proBNP was associated with greater risk, whereas the apparent attenuation of treatment effect at the highest concentrations remains hypothesis-generating and does not establish a validated treatment-selection threshold (Ezekowitz et al., 2020; Senni et al., 2022; Armstrong et al., 2022). Adjusted recurrent-event analysis of total HF hospitalizations and cardiovascular death was not statistically significant (HR 0.92, 95% CI 0.84–1.01); therefore, the primary time-to-first-event analysis remains the confirmatory efficacy evidence (Armstrong et al., 2020a; Mentz et al., 2024).

Symptomatic hypotension occurred in 9.1% versus 7.9% of patients receiving vericiguat and placebo, respectively, and anemia in 7.6% versus 5.7%; the modest early reduction in hemoglobin did not progress after 16 weeks or modify the treatment effect on the primary outcome (Armstrong et al., 2020a; Ezekowitz et al., 2021). VICTORIA therefore supports vericiguat in the represented clinically stabilized, recently worsening HF population; it did not evaluate treatment initiation during unstable acute decompensation (Armstrong et al., 2020a). Approximately 14% of participants had LVEF between 40% and 45%, creating partial overlap with the contemporary HFmrEF range, but VICTORIA was not a dedicated HFmrEF trial (McDonagh et al., 2021; Pieske et al., 2019; McDonagh et al., 2023).

### 6.3 Kidney function and CKD representation in VICTORIA

VICTORIA permitted baseline eGFR as low as 15 mL/min/1.73 m^2^ and excluded chronic dialysis (Armstrong et al., 2020a; Pieske et al., 2019). The renal-function study was *post hoc*, although the treatment-effect comparison in patients with eGFR ≤30 versus >30 mL/min/1.73 m^2^ was prespecified. Over 48 weeks, eGFR and serum creatinine trajectories were similar between vericiguat and placebo (interaction P = 0.50 and P = 0.18, respectively); the adjusted between-group difference in eGFR at week 48 was −0.40 mL/min/1.73 m^2^ (95% CI −1.49 to 0.70). Baseline eGFR did not significantly modify the treatment effect on the primary composite outcome. These findings do not establish renoprotection and should not be interpreted as proof of equal efficacy within each CKD stratum (Voors et al., 2021).

This represents kidney-function evidence derived from an HF trial rather than a dedicated CKD or HF–CKD kidney-outcome trial (Armstrong et al., 2020a; Voors et al., 2021). The analysis focused on renal-function measures rather than dedicated kidney outcomes, and its findings should not be extrapolated to patients with eGFR <15 mL/min/1.73 m^2^ or receiving dialysis (Pieske et al., 2019; Voors et al., 2021).

### 6.4 Stable ambulatory HFrEF: VICTOR

VICTOR enrolled 6,105 ambulatory patients with LVEF ≤40% without HF hospitalization within 6 months or outpatient intravenous diuretic treatment within 3 months before randomization. Contemporary background therapy included ARNI in 56.0% and SGLT2 inhibitors in 59.1% of participants. Over a median follow-up of 18.5 months, the primary composite of cardiovascular death or first HF hospitalization occurred in 18.0% with vericiguat and 19.1% with placebo (HR 0.93, 95% CI 0.83–1.04; P = 0.22); thus, the primary efficacy result was neutral (Butler et al., 2025).

Cardiovascular death, a prespecified key secondary endpoint, was lower with vericiguat (9.6% vs. 11.3%; HR 0.83, 95% CI 0.71–0.97), as was all-cause mortality (HR 0.84, 95% CI 0.74–0.97). However, because the primary endpoint was neutral, these mortality analyses used nominal P values and were not part of formal trial hypothesis testing; they therefore remain non-confirmatory (Butler et al., 2026). Although first HF hospitalization was not reduced (HR 0.95, 95% CI 0.82–1.10) (Butler et al., 2025), a subsequent exploratory analysis using a broader worsening-HF definition that also included urgent intravenous diuretic treatment and outpatient oral-diuretic initiation or intensification reported fewer events with vericiguat (686 vs. 748 participants; HR 0.90, 95% CI 0.81–1.00); the composite of all-cause death or expanded worsening HF yielded an HR of 0.90 (95% CI 0.82–0.98). These analyses were exploratory and were not adjusted for multiplicity; they therefore do not supersede the neutral primary result (Zannad et al., 2025a).

Symptomatic hypotension (11.3% vs. 9.2%) and anemia (7.6% vs. 6.3%) were more frequent with vericiguat (Butler et al., 2025). A prespecified pooled individual-participant analysis of VICTORIA and VICTOR favored vericiguat for cardiovascular death or first HF hospitalization (HR 0.91, 95% CI 0.85–0.98), but pooling does not supersede the neutral primary result of VICTOR (Zannad et al., 2025b). Accordingly, VICTOR does not support automatic extension of the worsening-HF indication to all stable HFrEF.

### 6.5 HFpEF and pulmonary-hypertension phenotypes

In SOCRATES-PRESERVED, 12 weeks of vericiguat did not improve either of the two primary endpoints, NT-proBNP or left atrial volume; a prespecified exploratory KCCQ-CSS signal was observed in the 10-mg arm (Pieske et al., 2017). In VITALITY-HFpEF, neither 15 nor 10 mg vericiguat improved the primary KCCQ-PLS endpoint versus placebo (LS mean differences −1.5 points, 95% CI −5.5 to 2.5; and −0.5 points, 95% CI −4.6 to 3.5, respectively), and the secondary 6-min walk distance was also neutral (Armstrong et al., 2020b). In CAPACITY HFpEF, praliciguat did not improve the primary peak oxygen-consumption endpoint (placebo-adjusted difference −0.30 mL/kg/min, 95% CI −0.95 to 0.35; P = 0.37) (Udelson et al., 2020). Together, these phase 2 trials do not demonstrate clinical benefit of sGC stimulation in broad HFpEF populations.

These neutral trials do not establish HFpEF heterogeneity as the explanation for treatment failure. HFpEF heterogeneity and abnormalities in NO–cGMP–PKG biology provide only a hypothesis-generating rationale for prospective enrichment, not validated predictors of sGC response. Future trials would need to prespecify phenotypic or biomarker-defined subgroups and test differential treatment response using clinically meaningful endpoints (van Heerebeek et al., 2012; Paulus and Tschöpe, 2013; Shah et al., 2015).

Riociguat studies define a narrower hemodynamic domain. In LEPHT, conducted in PH due to systolic LV dysfunction, the primary mPAP endpoint was not met (P = 0.10), although selected secondary hemodynamic measures improved (Bonderman et al., 2013). In PH-HFpEF, DILATE-1 did not meet its single-dose primary mPAP endpoint (P = 0.60), despite exploratory hemodynamic effects (Bonderman et al., 2014). DYNAMIC met its primary resting cardiac-output endpoint (LS mean difference 0.54 L/min, 95% CI 0.112–0.971; P = 0.0142), but did not improve clinical symptoms and led to more dropouts (Dachs et al., 2022). These studies demonstrate selected hemodynamic activity in PH phenotypes but do not establish clinical-outcome efficacy in HFpEF; none was designed to provide direct HF–CKD outcome evidence.

### 6.6 Albuminuria-focused CKD trials

In the phase 2a CONCORD trial, patients received stable maximum-tolerated RAS inhibition, with diabetic CKD strata defined by background SGLT2 inhibitor use. In the primary per-protocol analysis, runcaciguat reduced UACR versus placebo by 45.2% (95% CI, 32.3%–55.6%) without SGLT2 inhibition and by 48.1% (95% CI, 8.9%–70.5%) with SGLT2 inhibition; corresponding reductions in the full analysis set were similar (46.9% and 44.8%, respectively), whereas the difference was not significant in non-diabetic CKD. Modest blood-pressure lowering and a small reversible early eGFR reduction were also observed, so a hemodynamic contribution to the albuminuria effect cannot be excluded (Gansevoort et al., 2025).

In a prespecified pooled analysis of two 20-week phase 2b trials, avenciguat added to ACE inhibitor or ARB therapy produced placebo-corrected changes in the primary 10-h UACR endpoint of −15.5% (95% CI, −26.4 to −3.0), −13.2% (95% CI, −24.6 to −0.1), and −21.5% (95% CI, −31.7 to −9.8) with 1, 2, and 3 mg three times daily, respectively; 29% of participants were receiving an SGLT2 inhibitor at baseline. The short follow-up did not permit assessment of long-term eGFR change or clinical kidney outcomes (Heerspink et al., 2024).

In 156 adults with type 2 diabetes, CKD, and albuminuria receiving RAS inhibition, 12 weeks of praliciguat did not significantly reduce UACR in the primary efficacy analysis (placebo-corrected difference −15%, 90% CI −31% to 4%; P = 0.17) (Hanrahan et al., 2021).

Across the runcaciguat and avenciguat CKD activator programs, the recurring pattern is short-term UACR reduction over 8–20 weeks without demonstrated long-term eGFR preservation or benefit on clinical kidney or HF outcomes. The neutral praliciguat result cautions against assuming a uniform effect across sGC modulators; overall, these studies provide CKD-focused surrogate evidence rather than established HF–CKD outcome efficacy.

### 6.7 Cinaciguat and safety-limited acute-HF development

Intravenous cinaciguat produced marked hemodynamic unloading in acute decompensated HF, but substantial hypotension limited its development (Erdmann et al., 2013; Lapp et al., 2009). In a randomized phase 2b study, pulmonary capillary wedge pressure (PCWP) decreased by 7.7 mmHg with cinaciguat versus 3.7 mmHg with placebo at 8 h (P < 0.0001), while systolic blood pressure fell by 21.6 versus 5.0 mmHg; the trial was terminated early because of excess hypotension at higher doses (Erdmann et al., 2013). The subsequent low-dose COMPOSE program was also terminated early and did not establish symptomatic or clinical efficacy (Gheorghiade et al., 2012). Cinaciguat therefore defines a safety-limited translational boundary in which hemodynamic activity did not yield a clinically useful therapeutic window; these acute-HF studies do not constitute direct HF–CKD outcome evidence (Erdmann et al., 2013; Gheorghiade et al., 2012; Lapp et al., 2009).

### 6.8 Integrated clinical interpretation

The clinical evidence remains heterogeneous in endpoint strength and relevance to HF–CKD. VICTORIA demonstrated a reduction in the composite of cardiovascular death or first HF hospitalization after recent worsening HFrEF, driven predominantly by fewer HF hospitalizations, whereas VICTOR was neutral for the primary composite in stable ambulatory HFrEF (Armstrong et al., 2020a; Butler et al., 2025). Recent 2026 meta-analyses incorporating VICTOR yielded directionally consistent effect estimates, although statistical significance varied across analyses: HF phenotype-stratified analyses by Batista et al. and Kilic et al. showed a significant reduction in the composite of cardiovascular death or HF hospitalization among patients with HFrEF, whereas Nasir et al., using an overall HF population analysis, reported only a borderline non-significant reduction. These differences may reflect pooling across phenotypes, choice of effect measure (OR, RR, or HR), inclusion of phase 2 trials not powered for clinical events, and heterogeneity in baseline risk, follow-up, and background therapy; all were aggregate-level syntheses and therefore contextualize rather than supersede the primary results of VICTORIA and VICTOR (Batista et al., 2026; Nasir et al., 2026; Kilic et al., 2026).

Neutral HFpEF trials, riociguat hemodynamic studies, short-term CKD UACR trials, and hypotension-limited cinaciguat development did not establish HF–CKD clinical-outcome efficacy (Udelson et al., 2020; Gansevoort et al., 2025; Heerspink et al., 2024; Erdmann et al., 2013; Gheorghiade et al., 2012; Pieske et al., 2017; Armstrong et al., 2020b; Bonderman et al., 2013; Bonderman et al., 2014; Dachs et al., 2022). Direct clinical-outcome evidence specifically in HF–CKD remains limited: vericiguat evidence derives from HF trials, including a *post hoc* kidney-function analysis of VICTORIA, whereas runcaciguat and avenciguat were studied in CKD trials centered on albuminuria rather than HF outcomes (Gansevoort et al., 2025; Heerspink et al., 2024; Armstrong et al., 2020a; Voors et al., 2021; Butler et al., 2025). Table 2 summarizes these programs according to evidence context, efficacy, safety, and developmental status.

**TABLE 2:** Key human studies of sGC stimulators and activators across heart failure and chronic kidney disease phenotypes.

Study/agent	Design/population	Primary finding	Key background therapy	Key safety signal	Regulatory/development status	Evidence boundary
HFrEF/worsening-HF evidence						
SOCRATES-REDUCED (Gheorghiade et al., 2015)	Phase 2b; N = 456; stabilized worsening HFrEF, LVEF <45%; 12 weeks	Primary NT-proBNP analysis neutral; prespecified secondary dose-response signal exploratory	—	10-mg group: symptomatic hypotension 11.0% vs. 5.4% placebo	Vericiguat approved (US) for labeled recent-worsening chronic HF, LVEF <45% (Merck Sharp & Dohme LLC, 2026)	Dose selection/feasibility; not clinical-outcome efficacy
VICTORIA (Armstrong et al., 2020a)	Phase 3; N = 5,050; symptomatic HF, LVEF <45%, recent worsening; median 10.8 months	Primary composite reduced; benefit driven predominantly by fewer HF hospitalizations; CV death alone not significantly reduced	ACEi/ARB 73.5%; β-blocker 93.1%; MRA 70.4%; ARNI 14.5%; triple therapy 59.8%; SGLT2i uncommon	Symptomatic hypotension 9.1% vs. 7.9% (adjusted HR 1.17, 95% CI 0.96–1.42); anemia 7.6% vs. 5.7% (Lam et al., 2021b)	Approved (US) for symptomatic chronic HF, LVEF <45%, after recent worsening (Merck Sharp & Dohme LLC, 2026)	HF outcome evidence after recent worsening; not CKD-specific efficacy or renoprotection
VICTORIA kidney analysis (Voors et al., 2021)	Post hoc kidney-function analysis; N = 4,956; baseline eGFR ≥15 mL/min/1.73 m^2^; no dialysis; renal trajectories to 48 weeks	eGFR and creatinine trajectories similar; baseline eGFR did not significantly modify the primary treatment effect	As in VICTORIA.	Safety differences by eGFR reflected renal risk; no clear vericiguat-placebo excess within strata	No CKD indication; vericiguat label remains HF-specific (Merck Sharp & Dohme LLC, 2026)	Supports CKD representation/tolerability, not renoprotection or a kidney-outcome claim
VICTOR (Butler et al., 2025)	Phase 3; N = 6,105; LVEF ≤40%; stable ambulatory HFrEF without recent worsening; median 18.5 months	Primary composite neutral; mortality findings were secondary and non-confirmatory	β-blocker 94.4%; MRA 77.7%; RAS modulation 94.3% (including ARNI 56.0%); SGLT2i 59.1%	Symptomatic hypotension 11.3% vs. 9.2% (adjusted HR 1.24, 95% CI 1.06–1.46); anemia 7.6% vs. 6.3% (Mentz et al., 2025)	Current vericiguat label remains the recent-worsening HF indication (Merck Sharp & Dohme LLC, 2026)	Primary endpoint neutral; pooled analyses do not supersede this result
HFpEF and pulmonary-hypertension phenotypes						
SOCRATES-PRESERVED (Pieske et al., 2017)	Phase 2b; N = 477; worsening HFpEF; 12 weeks	Co-primary NT-proBNP and left-atrial-volume endpoints neutral; KCCQ-CSS signal exploratory	—	No clear excess in overall AEs; no meaningful BP difference at 10 mg vs. placebo	Vericiguat has no approved HFpEF indication	Does not establish clinical benefit in broad HFpEF.
VITALITY-HFpEF (Armstrong et al., 2020b)	Phase 2b; N = 789; HFpEF after decompensation; 24 weeks	Primary KCCQ-PLS endpoint neutral; 6MWD also neutral	—	Symptomatic hypotension: 6.4% (15 mg), 4.2% (10 mg), 3.4% placebo	Vericiguat has no approved HFpEF indication	Neutral health-status/functional evidence
CAPACITY HFpEF (Udelson et al., 2020)	N = 196 randomized; exercise-limited HFpEF; 12 weeks	Primary peak VO_2_ endpoint neutral	—	Hypotension 8.8% vs. 0%; AE discontinuation 5.5% vs. 3.3%	Praliciguat: investigational; phase 2 FSGS trial (NCT07268638) recruiting (Akebia Therapeutics, 2026)	Primary endpoint neutral; does not support praliciguat for unselected HFpEF.
LEPHT (Bonderman et al., 2013)	Phase 2b; N = 201; PH due to systolic LV dysfunction; 16 weeks	Primary mPAP endpoint neutral; selected secondary hemodynamic measures improved	Optimized HF therapy	Generally tolerated; discontinuation similar to placebo	Riociguat approved (US) for PAH/CTEPH, not left-heart PH.	Hemodynamic activity only; no clinical-outcome efficacy
DILATE-1 (Bonderman et al., 2014)	Phase 2a; N = 39; PH-HFpEF; single-dose study	Primary mPAP endpoint neutral; selected exploratory hemodynamic effects	—	Single-dose study; 2-mg dose reduced SBP by ∼12 mmHg vs. placebo	Same as LEPHT.	Acute pharmacodynamic evidence only
DYNAMIC (Dachs et al., 2022)	Phase 2b; N = 114; PH-HFpEF; 26 weeks	Resting cardiac output improved; clinical symptoms unchanged	—	Hypotension 22.4% vs. 12.5%; premature discontinuation 31.0% vs. 14.3%	Same as LEPHT.	Hemodynamic improvement did not establish clinical-outcome benefit
Albuminuria-focused CKD programs						
CONCORD/runcaciguat (Gansevoort et al., 2025)	Phase 2a; N = 243; CKD with T2D and/or hypertension; 8 weeks	Short-term UACR reduction in diabetic strata; non-diabetic comparison neutral	Stable maximum-tolerated ACEi/ARB; with/without SGLT2i; aldosterone antagonists excluded	TEAEs 69.0% vs. 52.5%; serious TEAEs were similar between groups	Runcaciguat: investigational; CONCORD phase 2 completed; no approved HF/CKD indication	Short-term surrogate signal; no durable kidney or HF outcome efficacy
Avenciguat pooled trials (Heerspink et al., 2024)	Two phase 2b trials; 504 randomized/500 treated; diabetic and non-diabetic CKD; 20 weeks	Short-term UACR reduction across tested doses	ACEi/ARB background; 29% on SGLT2i	Hypotension 6%/10%/8% vs. 3% placebo; AE discontinuation 4%/9%/9% vs. 3% placebo	Avenciguat: investigational; phase 2 CKD trials completed; no approved HF/CKD indication	Short-term UACR evidence does not establish long-term eGFR or clinical kidney/HF benefit
Praliciguat DKD (Hanrahan et al., 2021)	Phase 2; N = 156; T2D, CKD, albuminuria; 12 weeks	Primary UACR endpoint neutral	RAS inhibition in all; SGLT2i 7%	Any AE 42% vs. 44%; higher-dose arm had more serious AEs, discontinuations, and BP-lowering events	Praliciguat: investigational; phase 2 FSGS trial (NCT07268638) recruiting (Akebia Therapeutics, 2026)	Primary kidney surrogate endpoint neutral
Acute-HF safety boundary						
Cinaciguat acute-HF program (Erdmann et al., 2013; Gheorghiade et al., 2012; Lapp et al., 2009)	Phase 2/2b; IV cinaciguat in acute decompensated HF.	Hemodynamic unloading; subsequent low-dose COMPOSE did not establish symptomatic or clinical efficacy	Standard acute-HF therapy	Hypotension 73.2% vs. 25.5%; SBP change −21.6 vs. −5.0 mmHg; development limited by excess hypotension	No approved indication; acute-HF clinical development halted	Marked hemodynamic activity did not yield a clinically useful therapeutic window

No dedicated clinical-outcome trial in an explicitly defined HF-CKD population is represented here; preclinical combined cardiorenal evidence is summarized in Section 5 and Table 1. SK-08 has only phase 1 healthy-volunteer PK/PD and safety data and is therefore not included among the disease-population studies summarized here (Liu et al., 2026). Safety metrics are study-specific and are not directly comparable across trials. Reported comparative safety estimates are provided where available; NNH was not estimated because follow-up duration, dose structure, and adverse-event ascertainment differed substantially across programs.

Abbreviations: ACEi, angiotensin-converting enzyme inhibitor; ARB, angiotensin II receptor blocker; AE, adverse event; ARNI, angiotensin receptor–neprilysin inhibitor; BP, blood pressure; CI, confidence interval; CKD, chronic kidney disease; CTEPH, chronic thromboembolic pulmonary hypertension; CV, cardiovascular; DKD, diabetic kidney disease; eGFR, estimated glomerular filtration rate; FSGS, focal segmental glomerulosclerosis; HF, heart failure; HFpEF, heart failure with preserved ejection fraction; HFrEF, heart failure with reduced ejection fraction; HR, hazard ratio; IV, intravenous; KCCQ-CSS, Kansas City Cardiomyopathy Questionnaire Clinical Summary Score; KCCQ-PLS, Kansas City Cardiomyopathy Questionnaire Physical Limitation Score; LV, left ventricle/left ventricular; LVEF, left ventricular ejection fraction; mPAP, mean pulmonary artery pressure; MRA, mineralocorticoid receptor antagonist; NT-proBNP, N-terminal pro-B-type natriuretic peptide; PAH, pulmonary arterial hypertension; PH, pulmonary hypertension; PK/PD, pharmacokinetics/pharmacodynamics; RAS, renin–angiotensin system; SBP, systolic blood pressure; sGC, soluble guanylate cyclase; SGLT2i, sodium–glucose cotransporter 2 inhibitor; T2D, type 2 diabetes; TEAE, treatment-emergent adverse event; UACR, urinary albumin-to-creatinine ratio; VO_2_, oxygen consumption; 6MWD, 6-min walk distance.

## 7 Four-axis framework for interpreting sGC evidence in HF–CKD

### 7.1 HF phenotype and clinical state

The first axis is HF phenotype and clinical state. Recently worsening symptomatic HF, stable ambulatory HFrEF, HFmrEF, HFpEF, and pulmonary-hypertension phenotypes should not be treated as interchangeable. A recent worsening event identifies a high-risk clinical state (Lam et al., 2021a), but treatment effects should remain anchored to the population and endpoint directly studied; secondary biomarker or recurrent-event analyses do not independently establish efficacy (Ezekowitz et al., 2020; Senni et al., 2022; Armstrong et al., 2022; Mentz et al., 2024).

HFmrEF remains a specific evidence gap. Although the LVEF <45% criterion in VICTORIA partially overlapped with the contemporary HFmrEF range, VICTORIA was not a dedicated HFmrEF trial and does not provide dedicated efficacy evidence for HFmrEF (Heidenreich et al., 2022; Armstrong et al., 2020a; Pieske et al., 2019).

### 7.2 Kidney phenotype using KDIGO CGA

The second axis is CKD phenotype. KDIGO CGA distinguishes the cause of CKD, GFR category, and albuminuria category, with eGFR and albuminuria providing complementary prognostic information for kidney and cardiovascular risk (Stevens et al., 2024; Chronic Kidney Disease Prognosis Consortium et al., 2010; Gansevoort et al., 2011). Proteinuric CKD represented in short-term UACR trials should not be treated as equivalent to non-proteinuric CKD, G5 without dialysis, or G5D. Figure 1 maps selected CKD phenotype categories represented in the current evidence base and separates G5 from G5D as distinct evidence gaps. In this review, KDIGO CGA is therefore used to define the evidence context and its limits, rather than to infer differential response to sGC modulation.

**FIGURE 1:**
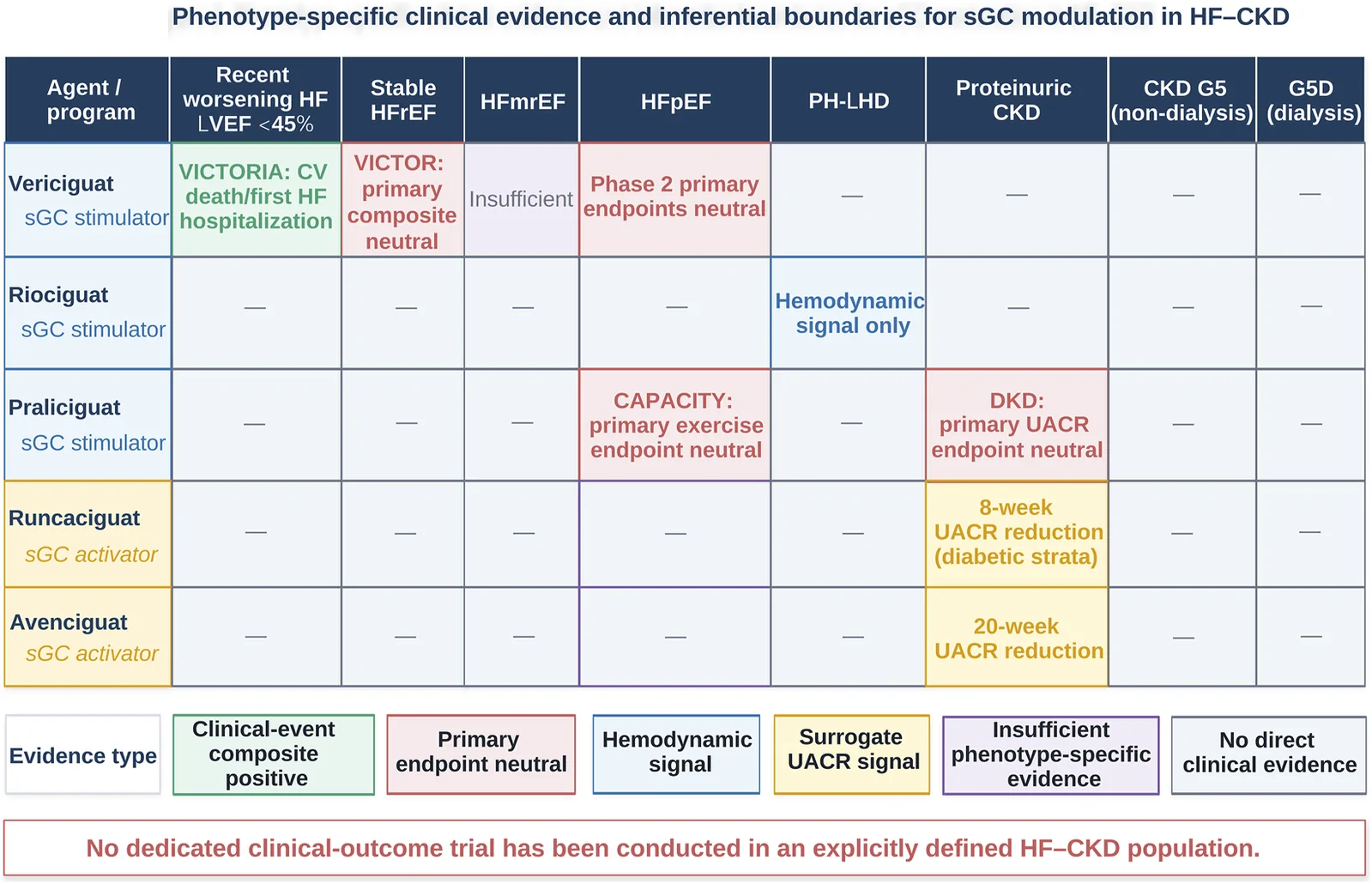
Phenotype-specific clinical evidence and inferential boundaries for sGC modulation across heart failure and chronic kidney disease contexts. This matrix summarizes human clinical evidence for sGC stimulators and activators across predefined HF and CKD phenotypes. Evidence categories indicate clinical-event composite benefit, primary endpoint neutral, hemodynamic signal, surrogate UACR signal, insufficient phenotype-specific evidence, or no direct clinical evidence. Insufficient phenotype-specific evidence indicates that available data are inadequate to support conclusions within a specific phenotype, whereas no direct clinical evidence indicates the lack of phenotype-specific human clinical evaluation. No dedicated clinical-outcome trial has been conducted in an explicitly defined HF–CKD population; therefore, evidence from HF-only or CKD-only populations should not be extrapolated to unstudied HF–CKD phenotypes. CKD G5 and G5D remain distinct evidence gaps and should not be inferred from proteinuric CKD populations. Cinaciguat and SK-08 are not included because their human evidence derives from acute heart failure and healthy-volunteer settings, respectively, which fall outside the HF–CKD phenotype matrix presented here. Abbreviations: CKD, chronic kidney disease; CV, cardiovascular; DKD, diabetic kidney disease; G5, KDIGO GFR category 5; G5D, dialysis-dependent CKD G5; GFR, glomerular filtration rate; HF, heart failure; HF–CKD, heart failure–chronic kidney disease; HFmrEF, heart failure with mildly reduced ejection fraction; HFpEF, heart failure with preserved ejection fraction; HFrEF, heart failure with reduced ejection fraction; KDIGO, Kidney Disease: Improving Global Outcomes; LVEF, left ventricular ejection fraction; PH-LHD, pulmonary hypertension due to left heart disease; sGC, soluble guanylate cyclase; UACR, urinary albumin-to-creatinine ratio.

### 7.3 Putative sGC redox state

The third axis is putative sGC redox biology. Reduced, heme-containing sGC is preferentially engaged by stimulators, whereas oxidized or heme-free sGC is engaged by activators (Derbyshire and Marletta, 2012; Liu et al., 2021; Evgenov et al., 2006; Follmann et al., 2013). This biochemical distinction is useful for drug discovery and preclinical interpretation, but as a clinical stratification axis, redox biology remains hypothesis-generating rather than validated. As detailed in Section 3.3, no validated clinical assay quantifies the relevant tissue sGC redox state, and no prospective treatment-by-biomarker interaction has validated redox-guided selection between stimulators and activators. Figure 2 places this mechanistic distinction within the broader evidence-interpretation framework.

**FIGURE 2:**
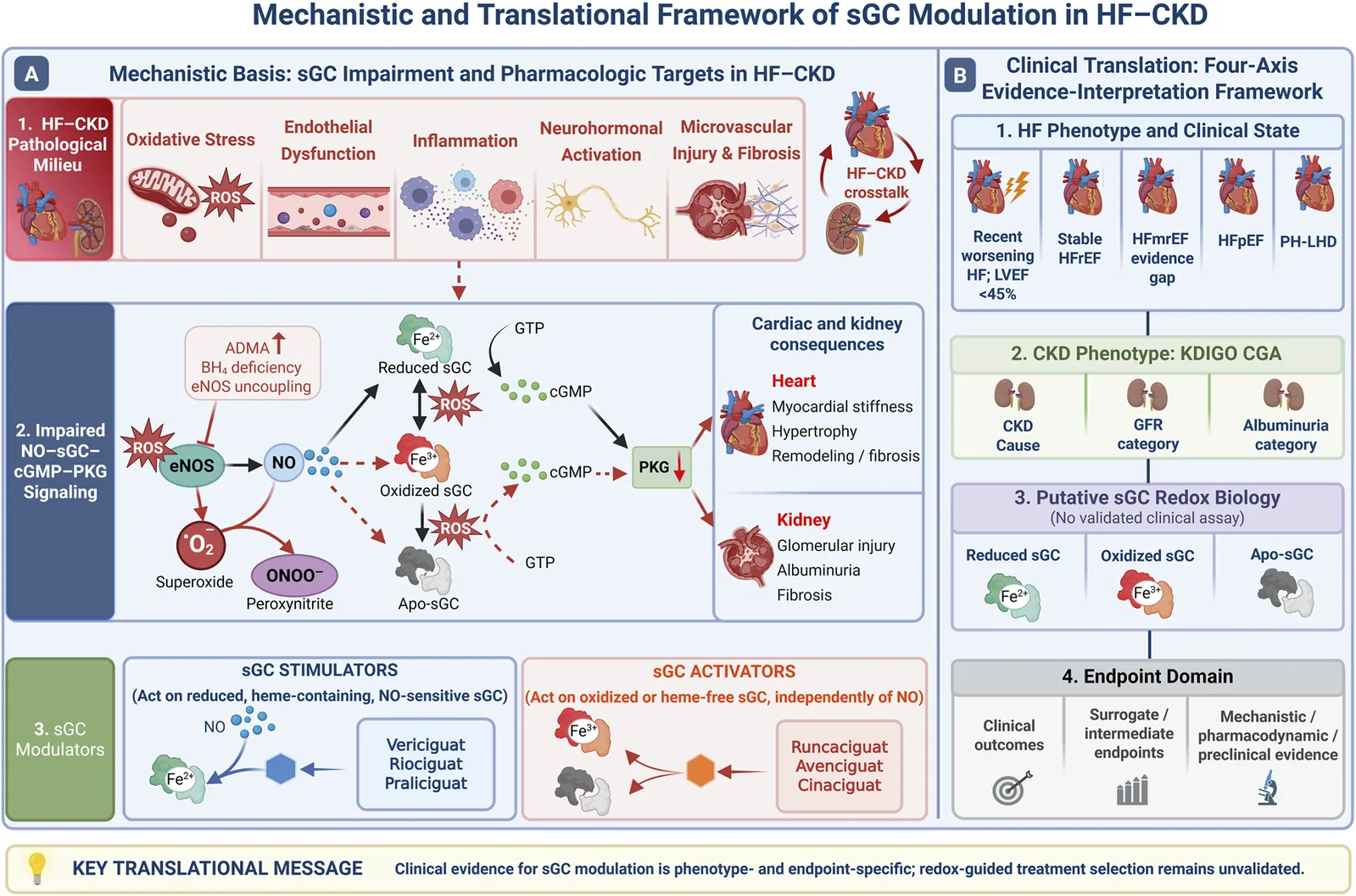
Mechanistic and translational framework of sGC modulation in HF–CKD. **(A)** The mechanistic framework illustrates how HF–CKD pathological processes, including oxidative stress, endothelial dysfunction, inflammation, neurohormonal activation, and microvascular injury/fibrosis, impair NO–sGC–cGMP–PKG signaling and contribute to downstream cardiac and renal consequences. Pharmacologic modulation of sGC includes stimulators that enhance signaling through reduced, heme-containing sGC and activators that engage oxidized or heme-free sGC independently of NO. **(B)** The clinical translation framework integrates four interpretive axes for sGC modulation in HF–CKD: HF phenotype and clinical state, CKD phenotype according to KDIGO CGA classification, putative sGC redox biology, and endpoint domain. Redox-guided treatment selection remains investigational because no validated clinical assay currently defines sGC redox state for therapeutic stratification. Clinical evidence should therefore be interpreted according to phenotype and endpoint hierarchy rather than extrapolated across unstudied populations. Abbreviations: ADMA, asymmetric dimethylarginine; BH_4_, tetrahydrobiopterin; CKD, chronic kidney disease; CGA, cause, GFR category, and albuminuria category; cGMP, cyclic guanosine monophosphate; eNOS, endothelial nitric oxide synthase; GFR, glomerular filtration rate; GTP, guanosine triphosphate; HF, heart failure; HF–CKD, heart failure–chronic kidney disease; HFmrEF, heart failure with mildly reduced ejection fraction; HFpEF, heart failure with preserved ejection fraction; HFrEF, heart failure with reduced ejection fraction; KDIGO, Kidney Disease: Improving Global Outcomes; LVEF, left ventricular ejection fraction; NO, nitric oxide; ONOO^−^, peroxynitrite; apo-sGC, heme-free (apo) soluble guanylate cyclase; PH-LHD, pulmonary hypertension due to left heart disease; PKG, protein kinase G; ROS, reactive oxygen species; sGC, soluble guanylate cyclase.

### 7.4 Endpoint domain

The fourth axis is the endpoint domain. Cardiovascular death and HF hospitalization are clinical event outcomes, whereas sustained eGFR decline and kidney failure are clinical kidney outcomes. UACR is an intermediate or surrogate measure whose interpretation depends on the magnitude and persistence of change, baseline albuminuria and eGFR, duration of follow-up, and supporting clinical-trial evidence (Gansevoort et al., 2011; Coresh et al., 2019). A short-term UACR reduction should therefore not be treated as equivalent to sustained preservation of kidney function or prevention of kidney failure.

NT-proBNP is a prognostic biomarker rather than a clinical outcome; secondary or *post hoc* VICTORIA analyses do not establish a validated biomarker threshold for treatment selection (Ezekowitz et al., 2020; Senni et al., 2022; Armstrong et al., 2022). Hemodynamics, cGMP, imaging, histology, and transcriptomics remain pharmacodynamic or mechanistic domains. These levels of evidence should be interpreted separately rather than collapsed into a single “cardiorenal benefit” claim.

Table 3 integrates these endpoint distinctions with the phenotype-specific evidence boundaries described above, specifying what can and cannot be inferred from each evidence context.

**TABLE 3:** Evidence domains and interpretive boundaries across heart failure and chronic kidney disease phenotypes.

Clinical phenotype/setting	Direct evidence domain	What can be stated	What must not be stated	Priority evidence gap
Recently worsening symptomatic HF; LVEF <45%; non-dialysis eGFR ≥15	Phase 3 RCT with a clinical-event composite endpoint	Vericiguat reduced the CV death/first HF hospitalization composite, driven mainly by fewer HF hospitalizations	Do not claim CV-mortality benefit, replacement of foundational GDMT, CKD-specific efficacy, or renoprotection	Contemporary-therapy interactions; advanced CKD representation
Stable ambulatory HFrEF without recent worsening	Phase 3 RCT with neutral primary endpoint	The primary composite was not significantly reduced	Do not present nominal mortality findings as confirmatory	Risk-enrichment and replication
HFmrEF	Indirect overlap within the VICTORIA LVEF <45% criterion	VICTORIA partially overlapped the contemporary HFmrEF range	Do not assume equivalence to HFrEF or HFpEF	Dedicated or prespecified LVEF-stratified evidence
HFpEF	Multiple neutral phase 2 biomarker/functional trials	Population-wide benefit has not been demonstrated	Do not attribute neutral results solely to insensitive endpoints or recommend routine use	Biologically enriched phenotypes with validated outcomes
PH-HFpEF/PH due to left-heart disease	Hemodynamic phase 2 studies	Riociguat can alter selected hemodynamics in defined PH phenotypes	Do not extrapolate to routine HFpEF or clinical-outcome efficacy	Larger outcome and safety studies
Proteinuric CKD, generally G2–G4/A2–A3	Phase 2 surrogate-endpoint RCTs	Runcaciguat and avenciguat lowered UACR over short follow-up	Do not claim sustained eGFR, kidney-failure, HF, or mortality benefit	Longer kidney-outcome trials on contemporary therapy
CKD G5 not receiving dialysis	No direct clinical evidence	No phenotype-specific clinical evidence is available	Do not merge with G5D or extrapolate from eGFR ≥15	Pharmacokinetics, hypotension, and outcomes
CKD G5D/dialysis	No direct efficacy evidence	Only the evidence gap can be stated	Do not infer dosing, dialyzability, or benefit	Dedicated dialysis studies
Redox-biomarker-selected HF–CKD	No prospective biomarker-selected clinical evidence; mechanistic/preclinical rationale only	Redox biology is a testable enrichment hypothesis	Do not use ADMA, cGMP, or oxidative markers as validated treatment-selection tests	Assay validation and prospective treatment interaction

Abbreviations: A1–A3, KDIGO albuminuria categories; ADMA, asymmetric dimethylarginine; CGA, cause–GFR–albuminuria; cGMP, cyclic guanosine monophosphate; CKD, chronic kidney disease; CV, cardiovascular; eGFR, estimated glomerular filtration rate; G2–G5, KDIGO glomerular filtration rate categories 2-5; G5D, dialysis-dependent CKD G5; GDMT, guideline-directed medical therapy; HF, heart failure; HFmrEF, HF with mildly reduced ejection fraction; HFpEF, HF with preserved ejection fraction; HFrEF, HF with reduced ejection fraction; LVEF, left ventricular ejection fraction; PH, pulmonary hypertension; RCT, randomized controlled trial; UACR, urinary albumin-to-creatinine ratio.

## 8 Evidence-informed clinical positioning and safety considerations

### 8.1 Directly represented population

The population most directly represented by positive clinical-outcome evidence is adults with symptomatic chronic HF, LVEF <45%, and a recent worsening-HF event, as studied in VICTORIA (Armstrong et al., 2020a; Merck Sharp & Dohme LLC, 2026). This evidence supports positioning vericiguat as an adjunct within this recent-worsening-HF setting.

VICTORIA permitted baseline eGFR as low as 15 mL/min/1.73 m^2^ (Armstrong et al., 2020a; Voors et al., 2021). This establishes representation of patients with impaired kidney function within an HF trial, but does not constitute CKD-specific efficacy evidence or establish kidney protection.

### 8.2 Clinical settings without established efficacy

The neutral primary result of VICTOR does not support extrapolating the VICTORIA evidence to stable ambulatory HFrEF without recent worsening; its mortality findings remain secondary and non-confirmatory (Butler et al., 2025). Neutral phase 2 studies likewise do not establish efficacy of sGC stimulation in broad HFpEF (Udelson et al., 2020; Pieske et al., 2017; Armstrong et al., 2020b). Selected hemodynamic effects of riociguat in pulmonary-hypertension phenotypes do not establish clinical-outcome efficacy in general HFpEF (Bonderman et al., 2013; Bonderman et al., 2014; Dachs et al., 2022).

CKD G5 and G5D remain outside the represented vericiguat outcome evidence (Voors et al., 2021). VICTORIA also does not establish efficacy for initiation during hemodynamically unstable acute HF (Armstrong et al., 2020a). Short-term albuminuria findings with investigational sGC activators in CKD do not provide HF-outcome evidence (Gansevoort et al., 2025; Heerspink et al., 2024).

### 8.3 Dose and regulatory boundaries

The current U.S. prescribing information, revised in January 2026, recommends vericiguat 5 mg orally once daily with food as the starting dose; for patients at risk of symptomatic hypotension, the recommended starting dose is 2.5 mg once daily with food. The dose is doubled approximately every 2 weeks to the target maintenance dose of 10 mg once daily, as tolerated (Merck Sharp & Dohme LLC, 2026).

This differs from the original VICTORIA protocol, in which vericiguat was initiated at 2.5 mg once daily and titrated to 10 mg (Armstrong et al., 2020a). Accordingly, clinical dosing and regulatory eligibility should follow current jurisdiction-specific prescribing information and guidance rather than be inferred from original trial protocols alone.

### 8.4 Hypotension, anemia, interactions, and kidney function

Hypotension and anemia are the most common adverse reactions in the U.S. label. Concomitant use with another sGC stimulator is contraindicated, and concomitant PDE5 inhibition is not recommended because of hypotension risk. Pregnancy is contraindicated because of embryo–fetal toxicity, and breastfeeding is not recommended. No dose adjustment is recommended for non-dialysis patients with eGFR ≥15 mL/min/1.73 m^2^; vericiguat has not been studied in patients with eGFR <15 at treatment initiation, in those receiving dialysis, or in those with severe hepatic impairment (Merck Sharp & Dohme LLC, 2026). Safety profiles should not be generalized across sGC modulators; the major comparative safety signals are summarized in Table 2.

In HF–CKD, hypotension and kidney-function changes are often multifactorial. Congestion, overdiuresis with intravascular volume depletion, changes in renin–angiotensin system therapy, and other hemodynamic or treatment-related factors may contribute. A transient creatinine rise should be interpreted together with volume status, blood pressure, electrolytes, and clinical response, with subsequent management individualized to the clinical context (Mullens et al., 2009; Testani et al., 2010; Metra et al., 2012; Bergen and Spaak, 2025; Pecoits‐Filho et al., 2025).

### 8.5 Integration with contemporary therapy

Within the evidence-supported worsening-HF population, vericiguat should be positioned as an adjunct to foundational guideline-directed therapy, optimized as tolerated, rather than as a substitute for it. Congestion control, iron deficiency and anemia, blood-pressure reserve, adherence, and comorbidity management remain part of the broader treatment context (Beldhuis et al., 2022; Maddox et al., 2024; Heidenreich et al., 2022; McDonagh et al., 2021). Background therapy differed across the two phase 3 vericiguat trials: SGLT2 inhibitor use was uncommon and ARNI use limited in VICTORIA, whereas both were substantially more common in VICTOR. VICTOR provided a more contemporary background-therapy context, but its primary efficacy result was neutral (Armstrong et al., 2020a; Pieske et al., 2019; Butler et al., 2025).

For investigational sGC modulators, the CKD trials were generally conducted on background RAS blockade, while SGLT2 inhibitor use varied across studies (Hanrahan et al., 2021; Gansevoort et al., 2025; Heerspink et al., 2024). These data do not yet establish incremental clinical-outcome benefit on contemporary multidrug cardiorenal therapy. Detailed background-therapy exposure across the major studies is summarized in Table 2; future combination and add-on trial design is discussed in Section 9.4.

## 9 Future trial design

### 9.1 Biomarker evaluation and redox-enrichment hypotheses

Future studies should distinguish pathway engagement, prognosis, and treatment prediction. Plasma or urinary cGMP may indicate pharmacodynamic activity, whereas ADMA, NO metabolites, BH_4_/BH_2_ balance, and oxidative-stress markers may describe redox biology; however, these systemic measures do not directly define tissue-specific sGC redox state. NT-proBNP, imaging, congestion measures, eGFR slope, UACR, and tubular-injury markers characterize disease severity. None should be considered a predictive biomarker unless a prospective treatment-by-biomarker interaction is replicated (Carlström, 2021; Carlström et al., 2024; Zoccali, 2006; Förstermann and Münzel, 2006; Schulz et al., 2008; Cuello and Nikolaev, 2020; Ezekowitz et al., 2020; Senni et al., 2022; Armstrong et al., 2022).

### 9.2 Dedicated HF–CKD programs for activators

A dedicated activator program should enroll an explicitly defined HF–CKD population and prespecify HF phenotype, CKD cause, GFR category, albuminuria category, diabetes, blood-pressure reserve, anemia, and congestion. High albuminuria and oxidative-stress measures may be evaluated as candidate enrichment variables, but they do not define a validated activator-responsive phenotype. Randomization should be stratified by contemporary SGLT2 inhibitor and renin–angiotensin system therapy, with careful capture of orthostatic symptoms, volume status, hemoglobin, and kidney-function changes.

### 9.3 Cardiovascular and kidney outcome architecture

Cardiovascular and kidney domains should be prespecified and reported separately, including when combined in a composite endpoint. Relevant cardiovascular outcomes include cardiovascular death, first and total HF hospitalizations, and urgent HF visits, while symptoms and health status should be analyzed as distinct patient-centered outcomes. Relevant kidney endpoints include sustained eGFR decline, eGFR slope, kidney failure, and dialysis initiation. UACR can support dose selection and provide a surrogate kidney signal (Coresh et al., 2019), but claims of durable kidney benefit require adequately powered trials with prespecified clinical kidney outcomes and sufficient follow-up.

### 9.4 Combination therapy and adaptive safety optimization

Because sGC modulators will be added to multidrug regimens, combination studies should assess pharmacodynamic redundancy and cumulative hypotension. Factorial or add-on designs with SGLT2 inhibitors may be especially informative for CKD activators. Run-in periods, slower titration, adaptive dose selection, and prespecified low-blood-pressure strata can improve interpretability, but the studies should not exclude the multimorbid patients most relevant to practice.

### 9.5 Advanced CKD and dialysis

CKD G5 without dialysis and G5D require separate pharmacokinetic, safety, and outcome programs, with dialyzability assessed specifically in G5D. Dialysis introduces large volume shifts, intradialytic hypotension, altered protein binding, vascular calcification, arrhythmia, myocardial stunning, and competing modes of death. These features cannot be inferred from non-dialysis eGFR thresholds. Initial studies should establish exposure, timing relative to dialysis, and hemodynamic tolerability before efficacy trials.

### 9.6 HFpEF phenotyping and mechanistic trials

Future HFpEF trials should treat phenotypic heterogeneity as a prospective enrichment hypothesis rather than a *post hoc* explanation for neutral trials. Candidate enrichment variables may include obesity–metabolic phenotypes, pulmonary vascular/RV involvement, CKD-associated phenotypes, and microvascular dysfunction or NO–cGMP–PKG abnormalities, but none is currently a validated predictor of sGC response. Mechanistic studies may use biomarkers, cardiac imaging, exercise testing, and hemodynamic phenotyping; efficacy trials should prespecify clinically meaningful functional or clinical outcomes (van Heerebeek et al., 2012; Paulus and Tschöpe, 2013; Shah et al., 2015; Redfield et al., 2013).

## 10 Limitations of this review

Several limitations of this review should be acknowledged. First, this was a narrative rather than a systematic or scoping review. Although the literature search was updated through 29 August 2026 and used PubMed/MEDLINE, ClinicalTrials.gov, regulatory sources, and reference lists of key sources, no formal risk-of-bias assessment, certainty grading, or meta-analysis was performed. Accordingly, study selection and emphasis may be affected by publication and selection bias, and the synthesis still depends primarily on available published primary reports. Second, cross-study interpretation is constrained by substantial heterogeneity in HF and CKD phenotypes, background therapy, follow-up duration, and endpoint domains, while direct clinical-outcome evidence in explicitly defined HF–CKD populations remains limited. Third, several sGC development programs remain ongoing or rapidly evolving, so the evidence base may change. Finally, the four-axis framework is interpretive and hypothesis-generating rather than prospectively validated; no validated clinical assay defines tissue sGC redox state, and no prospective treatment-by-biomarker interaction has validated redox-guided treatment selection.

## 11 Conclusion

Impaired NO–sGC–cGMP–PKG signaling provides a compelling biological bridge between cardiac and kidney dysfunction, but the clinical effects of sGC modulation are drug- and phenotype-specific. In VICTORIA, vericiguat reduced the primary composite endpoint in recently worsening symptomatic HF with LVEF below 45%, driven predominantly by fewer HF hospitalizations; VICTOR was neutral for its primary endpoint in stable HFrEF; broad HFpEF trials were neutral; and investigational activators in CKD currently provide albuminuria signals rather than established kidney-failure or HF benefits.

The most defensible interpretation keeps four axes separate: HF phenotype, KDIGO CGA risk, sGC redox biology, and endpoint domain. The biochemical distinction between stimulators and activators is established, but redox-guided treatment selection is not. Mechanistic effects, cGMP changes, hemodynamics, UACR, and clinical outcomes must not be treated as interchangeable evidence.

Future development should prioritize phenotype-enriched, biomarker-informed trials on contemporary background therapy, with prespecified cardiovascular and kidney outcomes and dedicated programs for CKD G5 and G5D. Until then, current use should remain within direct trial and regulatory boundaries, and investigational findings should be described with their inferential limits.
