# Supplementary material for: Soluble guanylate cyclase modulation in heart failure and chronic kidney disease: mechanisms, clinical evidence, and translational boundaries
Source: Front Pharmacol. 2026 Oct 1;17:1954549. doi: 10.3389/fphar.2026.1954549 (PMC13668576; doi:10.3389/fphar.2026.1954549)
Supplement: Supplementary file 1 [file Supplementaryfile1.docx]

**Supplementary Search Strategy and Evidence-Handling Framework**

*Soluble Guanylate Cyclase Modulation in Heart Failure and Chronic Kidney Disease*

# Supplementary Table S1. Source-specific search strategies and final search dates

The searches were designed to support a transparent and updateable structured narrative review. They were not intended to constitute an exhaustive systematic-review search or to support a PRISMA flow analysis or quantitative synthesis.

## A. PubMed/MEDLINE

| **Platform** | PubMed |
| --- | --- |
| **Coverage** | Database inception to 29 August 2026 |
| **Final search date** | 29 August 2026 |
| **Notes** | No publication-date or study-design filter was applied. English-language full texts were eligible. |

**Search string**

(
 "soluble guanylate cyclase"[Title/Abstract]
 OR "soluble guanylyl cyclase"[Title/Abstract]
 OR sGC[Title/Abstract]
 OR vericiguat[Title/Abstract]
 OR riociguat[Title/Abstract]
 OR praliciguat[Title/Abstract]
 OR runcaciguat[Title/Abstract]
 OR avenciguat[Title/Abstract]
 OR cinaciguat[Title/Abstract]
 OR "BAY 1021189"[Title/Abstract]
 OR "BAY 63-2521"[Title/Abstract]
 OR "BAY 60-4552"[Title/Abstract]
 OR "BAY 58-2667"[Title/Abstract]
 OR "BAY 41-8543"[Title/Abstract]
 OR "BAY 60-2770"[Title/Abstract]
 OR "BI 685509"[Title/Abstract]
 OR HEC95468[Title/Abstract]
 OR "SK-08"[Title/Abstract]
)
AND
(
 "Heart Failure"[MeSH Terms]
 OR "heart failure"[Title/Abstract]
 OR HFrEF[Title/Abstract]
 OR HFpEF[Title/Abstract]
 OR HFmrEF[Title/Abstract]
 OR "worsening heart failure"[Title/Abstract]
 OR "Cardio-Renal Syndrome"[MeSH Terms]
 OR cardiorenal[Title/Abstract]
 OR "Renal Insufficiency, Chronic"[MeSH Terms]
 OR "chronic kidney disease"[Title/Abstract]
 OR CKD[Title/Abstract]
 OR albuminuria[Title/Abstract]
 OR proteinuria[Title/Abstract]
 OR "kidney failure"[Title/Abstract]
 OR dialysis[Title/Abstract]
 OR "pulmonary hypertension due to left heart disease"[Title/Abstract]
 OR (
 (
 "Nitric Oxide"[MeSH Terms]
 OR "nitric oxide"[Title/Abstract]
 OR cGMP[Title/Abstract]
 OR "protein kinase G"[Title/Abstract]
 OR PKG[Title/Abstract]
 OR redox[Title/Abstract]
 OR oxidized[Title/Abstract]
 OR "heme-free"[Title/Abstract]
 OR "apo-sGC"[Title/Abstract]
 OR endothelial[Title/Abstract]
 OR microvascular[Title/Abstract]
 OR podocyte*[Title/Abstract]
 OR fibrosis[Title/Abstract]
 OR remodeling[Title/Abstract]
 )
 AND
 (
 heart[Title/Abstract]
 OR cardiac[Title/Abstract]
 OR kidney[Title/Abstract]
 OR renal[Title/Abstract]
 )
 )
)
AND English[Language]

## B. ClinicalTrials.gov

| **Platform** | ClinicalTrials.gov |
| --- | --- |
| **Coverage** | All registered years available in the database |
| **Final search date** | 29 August 2026 |
| **Notes** | Registry records were classified by recruitment status and by whether results or a peer-reviewed publication were available. |

**Search string**

ClinicalTrials.gov was searched separately using each of the following intervention or other terms:

vericiguat; riociguat; praliciguat; runcaciguat; avenciguat; cinaciguat; BI 685509; BAY 58-2667; BAY 41-8543; BAY 60-2770; HEC95468; SK-08; soluble guanylate cyclase; soluble guanylyl cyclase.

The following conditions were reviewed:
heart failure; heart failure with reduced ejection fraction; heart failure with preserved ejection fraction; worsening heart failure; chronic kidney disease; cardiorenal syndrome; albuminuria; proteinuria; kidney failure; dialysis; pulmonary hypertension due to left heart disease.

Study type: interventional studies. Recruitment status: all statuses.

## C. U.S. Food and Drug Administration

| **Platform** | Drugs@FDA |
| --- | --- |
| **Coverage** | Current and archived regulatory records available through 29 August 2026 |
| **Final search date** | 29 August 2026 |
| **Notes** | Regulatory records were used for approved use, dosing, contraindications, and warnings, not as substitutes for primary efficacy reports. |

**Search string**

Search terms entered separately in Drugs@FDA:

vericiguat; VERQUVO; riociguat; ADEMPAS.

Fields reviewed:
approved indication; approval history; current prescribing information; starting dose; target dose; contraindications; warnings and precautions; renal impairment; hepatic impairment; pregnancy; drug interactions; adverse reactions.

## D. European Medicines Agency

| **Platform** | EMA medicines database |
| --- | --- |
| **Coverage** | Current and archived regulatory records available through 29 August 2026 |
| **Final search date** | 29 August 2026 |
| **Notes** | Regulatory records were used to clarify European regulatory status and labeling boundaries. |

**Search string**

Search terms entered separately in the European Medicines Agency medicines database:

vericiguat; Verquvo; riociguat; Adempas; soluble guanylate cyclase; soluble guanylyl cyclase.

Fields reviewed:
European public assessment report; approved indication; dose; contraindications; warnings; renal and hepatic impairment; pregnancy; interactions; adverse reactions; regulatory status.

## E. Citation searching

Backward citation searching was conducted from pivotal randomized trials, major guidelines, pharmacology reviews, and directly relevant preclinical studies. Forward citation searching was used for pivotal clinical trials and major drug-development reports. Citation-derived records were subjected to the same eligibility and evidence-classification criteria as database-derived sources.

# Supplementary Table S2. Eligibility, duplicate handling, and study-selection framework

| **Domain** | **Procedure** |
| --- | --- |
| Review designation | Structured narrative review; not a systematic review, scoping review, or meta-analysis. |
| Primary scope | Mechanistic, preclinical, pharmacological, clinical, and regulatory evidence on sGC modulation relevant to HF, CKD, and HF–CKD translation. |
| Human clinical inclusion | Randomized or non-randomized studies of sGC modulators; pivotal trial companion analyses; phase 1/PK-PD studies; directly relevant observational studies, guidelines, and meta-analyses. |
| Preclinical inclusion | Studies directly evaluating sGC modulators in cardiac, renal, or combined cardiorenal models or mechanisms. |
| Regulatory inclusion | FDA, EMA, and jurisdiction-specific prescribing information relevant to approved indication, dosing, contraindications, warnings, and development status. |
| Exclusions | No direct sGC relevance; no cardiac/renal/cardiorenal relevance; conference abstract or commentary without sufficient data; duplicate or superseded report without a distinct analysis; non-English full text not reliably evaluable; unstable or unverifiable source. |
| Language | English-language full-text reports. |
| Duplicate handling | DOI, PMID, NCT number, exact title, and author-year-title matching followed by manual review of companion and follow-up reports. |
| Selection process | Potentially eligible sources and final evidence classifications reviewed by WY and BZ; disagreements resolved by consensus, with CY adjudicating unresolved issues. |
| Multiple publications | Primary report retained as the confirmatory efficacy source; companion reports retained only when they contributed distinct kidney, biomarker, safety, recurrent-event, mortality, or pooled-analysis information. |
| Evidence weighting | Direct randomized clinical-outcome trials > prespecified secondary analyses > phase 1/2 surrogate or pharmacodynamic studies > observational/post hoc analyses > preclinical/mechanistic studies for clinical efficacy inference. |
| Formal appraisal | No formal study-level risk-of-bias instrument, certainty grading, protocol registration, PRISMA flow analysis, or quantitative meta-analysis. |

# Supplementary Table S3. Evidentiary handling of registry and regulatory sources

| **Source category** | **Permitted use in this Review** | **Interpretive boundary** |
| --- | --- | --- |
| Recruiting / not yet recruiting registry study | Development status, planned population, intervention, and endpoints | Not used to establish efficacy or safety. |
| Active, not recruiting registry study | Development status and protocol-defined objectives | Not used to establish efficacy or safety. |
| Completed registry study with posted results but no peer-reviewed article | Explicitly described as unpublished registry results; may identify emerging signals | Lower interpretive weight than peer-reviewed primary reports; no definitive efficacy claim. |
| Completed registry study without posted results | Identifies an evidence gap and possible publication bias | No efficacy or safety inference. |
| Terminated or withdrawn registry study | Development history and stated reason for termination/withdrawal when available | No efficacy inference; safety interpretation limited to publicly available information. |
| Peer-reviewed primary trial publication | Principal source for efficacy, safety, numerical estimates, and confirmatory endpoint interpretation | Primary evidentiary source within the represented population and endpoint. |
| Companion or subgroup publication | Kidney function, biomarkers, recurrent events, safety, background therapy, or subgroup context | Does not supersede the primary trial endpoint; post hoc findings remain exploratory. |
| FDA / EMA regulatory document | Approved indication, dose, contraindications, warnings, interactions, organ-impairment statements, and regulatory status | Not a substitute for peer-reviewed efficacy evidence. |
| Manufacturer prescribing information | Current jurisdiction-specific dosing and safety information | Used only when current and official; efficacy interpretation remains anchored to peer-reviewed trials. |

Note: Registry-only, ongoing, or unpublished studies were not used to establish efficacy. Regulatory documents were used to define approved indications, dosing, contraindications, warnings, and development status. Peer-reviewed primary trial reports were prioritized for efficacy and safety interpretation.
